# Reconstruction–hybridization of molecular and metallic interfaces for efficient oxygen evolution

**DOI:** 10.1039/d6sc02174c

**Published:** 2026-05-27

**Authors:** Fei Wei, Dongliang Zhang, Yinbo Zhan, Haijiao Lu, Guoqiang Shen, Peng Liu, Xufang Qian, Xia Long

**Affiliations:** a State Key Laboratory of Green Papermaking and Resource Recycling, China-UK Low Carbon College, Shanghai Jiao Tong University Shanghai 201306 China x.long@sjtu.edu.cn; b Nanomaterials Centre, School of Chemical Engineering, Australian Institute for Bioengineering and Nanotechnology, The University of Queensland St Lucia Queensland 4072 Australia; c College of Smart Energy, Shanghai Jiao Tong University 800 Dong Chuan Rd. Minhang District Shanghai 200240 China; d Biolin Trading Company Ltd Shanghai 201203 China; e State Key Laboratory of Green Papermaking and Resource Recycling, School of Environmental Science and Engineering, Shanghai Jiao Tong University Shanghai 200240 China

## Abstract

Electrochemical reconstruction of molecular catalysts offers a route to high-performance oxygen evolution reaction (OER) interfaces, but the roles of the molecular centre and substrate in this process remain unclear. Here, we employ a “reconstruction–hybridization” strategy using metal phthalocyanines (M_1_Pc) as molecular precursors and metal foils (M_2_) as catalytic hosts. In the FePc/Ni model system, *in situ* and *ex situ* characterization reveal that activation transforms Fe–N_4_ sites to generate a near-surface, disordered, oxygen-coordinated Fe-containing interface on the Ni substrate, which is distinct from bulk oxidation and hence enhances the OER performance by accelerating intermediate adsorption and charge transfer. Systematically screening across M_1_ and M_2_ (M_1_ and M_2_ = Fe, Co, Ni, Cu) establishes the design principle that M_1_ should possess appropriate redox properties to facilitate interfacial reconstruction while M_2_ can serve as an active host. The poor performance of the inverse system of NiPc/Fe further supports the non-interchangeable roles of M_1_ (modulator) and M_2_ (active host). This work provides mechanistic insight into dynamic interface formation and a general framework for designing efficient multi-metal OER catalysts *via* electrochemical reconstruction.

## Abstract 2

FePc/Ni undergoes electrochemical reconstruction into an oxygen-coordinated Fe-containing interface, accelerating oxygenated-intermediate adsorption and charge transfer for efficient OER.

## Graphical abstract

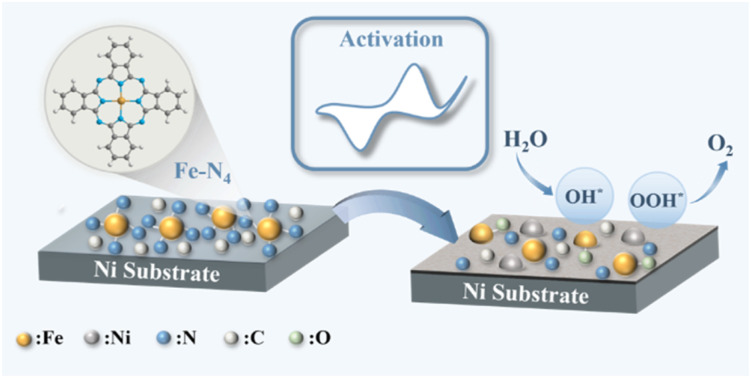

## Introduction

The rapidly increasing global energy demand and growing pressure from climate change are accelerating the development of renewable-based clean energy systems.^1–4^ Within this context, electrochemical water splitting has emerged as one of the most promising routes for green hydrogen production.^5–7^ However, the anodic oxygen evolution reaction (OER), involving multiple proton-coupled electron transfer (PCET) steps with intrinsically sluggish kinetics, remains the primary bottleneck limiting overall energy conversion efficiency.^8–10^ Therefore, the development of efficient, stable, and cost-effective OER catalysts is of critical importance.

Transition-metal-based catalysts, particularly those containing Ni and Fe, have attracted extensive attention due to their natural abundance and high intrinsic activity in alkaline media.^11–13^ It has been established that Ni/Fe (oxy)hydroxides can significantly modulate the binding energetics of key oxygenated intermediates through synergistic metal–metal interactions,^14,15^ thereby delivering outstanding OER performance. Indeed, such systems are widely regarded as the benchmark non-noble metal OER catalysts for practical applications.^16,17^ A now well-accepted paradigm is that most transition metal-based OER catalysts undergo surface reconstruction under operational conditions.^18–20^ Yet, fundamental questions persist regarding how this reconstruction unfolds dynamically and how the active components in the pre-catalysts interact with the metallic substrate (commonly nickel) to form the interfacial layer that ultimately dictates OER performance. These aspects remain poorly elucidated, largely because traditional multi-metal (oxy)hydroxides possess complex phase composition and ambiguous local environments that obscure the reconstruction pathway.^21,22^

Molecular complexes with well-defined structures offer a compelling alternative to circumvent these limitations. Metal phthalocyanines (MPcs), featuring a single, uniform M–N_4_ coordination centre, serve as ideal model pre-catalysts for probing reconstruction dynamics.^23–25^ Unlike deliberately engineered multi-metal oxides with ill-defined phase boundaries and mixed coordination motifs, MPcs offer structural simplicity that facilitates a more straightforward investigation of metal-site evolution during the OER, particularly at high anodic potentials. Although MPcs are known to irreversibly transform into active phases,^26,27^ most studies have merely confirmed the occurrence of reconstruction or identified the final phase.^28^ The dynamic interplay between molecular precursors and the substrate, and its role in dictating the nature and activity of the reconstructed interface, remain largely unexplored. This knowledge gap is especially consequential given that single-metal-site molecular catalysts often exhibit modest OER activity on their own.^29–31^ In contrast, extensive literature confirms that Fe–Ni coexistence in alkaline media leads to dramatically enhanced OER kinetics,^32–35^ suggesting that coupling a molecular Fe source with a Ni-based substrate could enable synergistic interfacial reconstruction. However, the dynamic evolution of molecular-substrate interactions during electrochemical activation, the formation mechanism of the reconstructed interfacial state, and the distinct roles of the molecular metal centre and the metallic substrate in this process remain poorly understood.

**Scheme 1:**
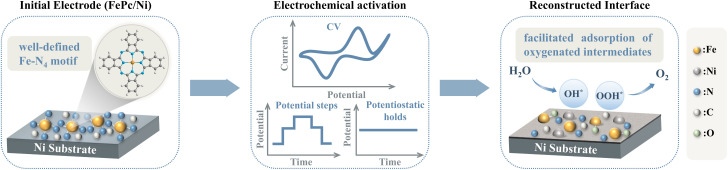
Schematic illustration for the formation of the FePc/Ni restructured interface.

In this work, we employed a “reconstruction–hybridization” strategy (Scheme 1) using FePc immobilized on Ni substrates (FePc/Ni) as a model system. By integrating *operando* electrochemical monitoring with complementary *in situ* and *ex situ* characterizations, we have tracked the transformation of molecular Fe–N_4_ sites into an amorphous, oxygen-coordinated Fe/Ni-containing reconstructed interface during OER activation. Our results demonstrated that reconstruction is not a bulk oxidation process but rather a spatially confined interfacial phenomenon driven by strong electronic coupling between Fe species derived from the molecular precursor and Ni species leached from the substrate. This reconstructed interface significantly enhanced OER kinetics by optimizing the adsorption strength of oxygenated intermediates and facilitating interfacial charge transfer. Extending this approach across a series of MPcs (M_1_Pc) and metallic substrates (M_2_), we established two general design principles for high-performance reconstructed catalysts: the molecular metal centre (M_1_) should effectively modulate the anodic transformation of the substrate during activation, while the M_2_ should provide a catalytically active framework capable of cooperative interaction with M_1_. Through rational pairing of precursors and substrates, our strategy enables the *in situ* construction of highly active multi-metallic interfacial sites, synergistically boosting both activity and reaction kinetics. This work thus provides mechanistic clarity on molecular-substrate coevolution during electrochemical reconstruction and offers a generalizable blueprint for the rational design of next-generation OER catalysts.

## Results and discussion

### Evolution of OER performance during electrochemical activation

FePc was deposited onto a Ni plate *via* spray-coating to fabricate the FePc/Ni electrode. Micro-attenuated total reflection Fourier transform infrared (micro-ATR-FTIR) spectroscopy showed pronounced peaks at ∼729, ∼1076, and ∼1119 cm^−1^ that were consistent with characteristic phthalocyanine macrocycle fingerprints,^36,37^ supporting the successful deposition of FePc on the Ni substrate (Fig. S1). Subsequently, cyclic voltammetry (CV), potentiostatic holds at selected potentials, and a multi-potential step protocol were carried out for electrode activation with the same total treatment time (Scheme 1, see the SI for details). Overall, among these activation protocols, CV cycling delivered the most favourable OER kinetics, as evidenced by the lowest Tafel slope (Fig. S2) and charge-transfer resistance (*R*_ct_, Fig. S3) together with competitive overpotentials across a broad current density window (Fig. S4 and S5), particularly at high current densities (Table S1). A plausible reason for this superior performance is that repeated potential cycling across the pre-OER redox region provides a more gradual and dynamically regulated driving force for interfacial reconstruction than potentiostatic or stepwise activation, thereby favouring the formation of a reconstructed interface with improved charge-transfer and catalytic properties. Accordingly, CV activation was selected as the standard procedure for all subsequent studies.

**Fig. 1:**
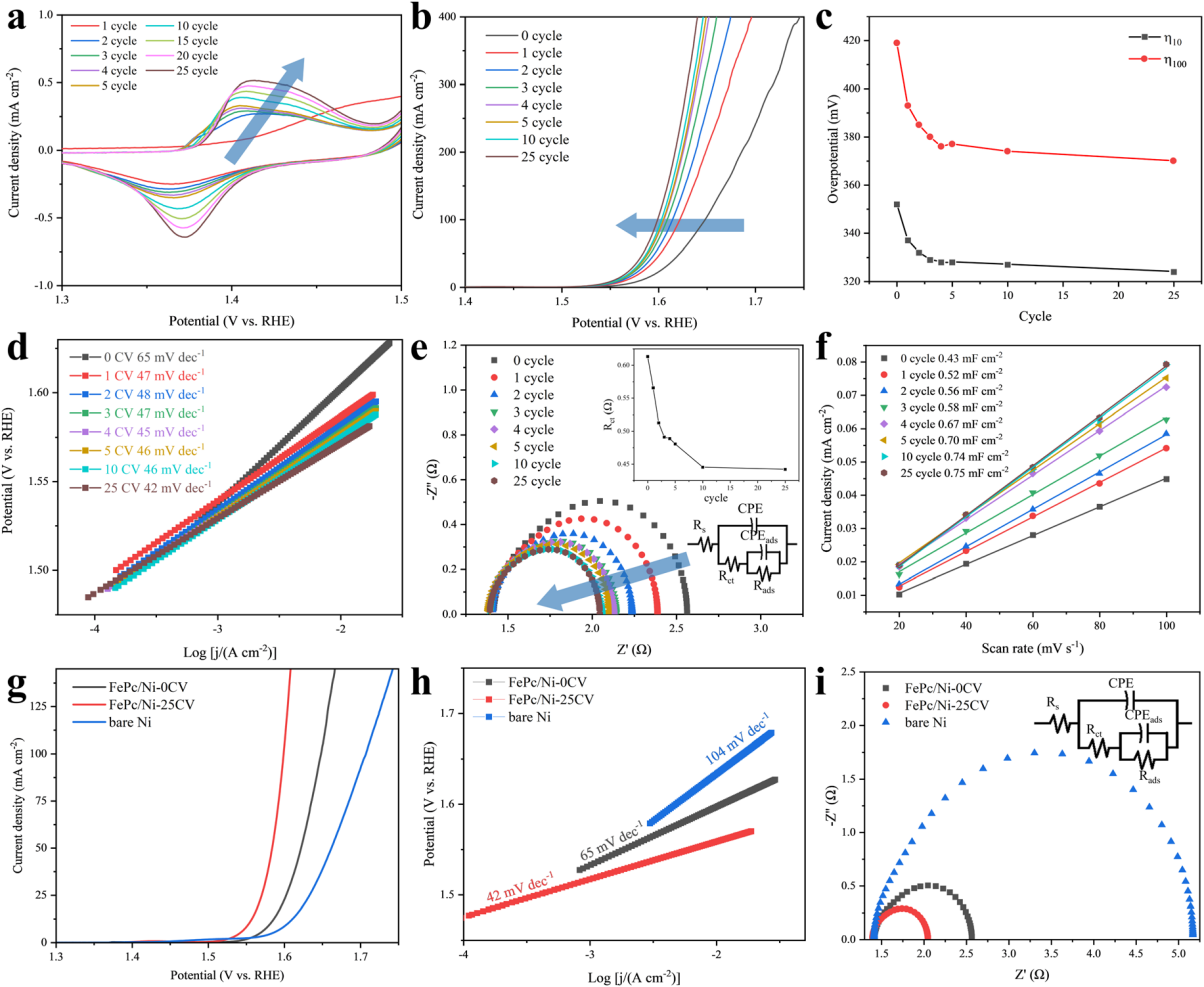
Evolution of the OER performance of FePc/Ni during CV activation. (a) CV curves at different activation cycles. (b) LSV curves, (c) overpotentials at specific current densities, (d) Tafel slope, (e) EIS spectra (inset: evolution of *R*_ct_, as a function of cycle number) and (f) double-layer capacitance recorded after different activation cycles. (g) LSV curves, (h) Tafel plots and (i) EIS spectra before/after activation and of bare Ni.

CV activation was employed to probe the electrochemical evolution of the FePc/Ni electrode. As shown in Fig. 1a, the broad pre-oxidation peak that could be assigned to the metal-site redox transition preceding OER onset progressively intensified during the initial CV cycles. Notably, both the position and intensity of this broad pre-oxidation peak approached a plateau by approximately 25 cycles (Fig. S6), suggesting that the surface activation process was largely complete by this point. To corroborate this observation, we evaluated the OER performance of electrodes activated for varying numbers of CV cycles. The overpotentials required to reach fixed current densities decreased sharply during the first 25 cycles: *η*_10_ decreased from 352 to 324 mV (Δ = 28 mV) and *η*_100_ decreased from 419 to 370 mV (Δ = 49 mV) (Fig. 1b and Table S2). Notably, the majority of the activity enhancement occurred during the early activation stage: by cycle 5, the overpotentials (*η*_10_ and *η*_100_) had already decreased by ∼7% and ∼10%, respectively. Only marginal improvements were observed in the later stages, as exemplified by *η*_100_ decreasing by only ∼1% from cycle 10 to 25 (Fig. 1c).

In parallel, the Tafel slope decreased from 65 to 42 mV dec^−1^ (Δ = 23 mV dec^−1^) (Fig. 1d and Table S2). As the activation approached 25 cycles, the changes became marginal and the trends levelled off, indicating that the catalytic activity and apparent reaction kinetics reached a steady state concurrent with stabilization of the pre-oxidation peak. Collectively, these convergent observations strongly suggested that the reconstructed surface structure and thus the catalytically relevant active phase were largely established by the 25th cycle. To further substantiate the establishment of a conductive and electrochemically accessible interface during activation, electrochemical impedance spectroscopy (EIS) was conducted after different numbers of CV cycles. *R*_ct_ decreased monotonically with cycling (Fig. 1e), indicating progressively facilitated interfacial charge transfer as reconstruction proceeds. In parallel, the double-layer capacitance (*C*_dl_) increased from 0.43 to 0.75 mF cm^−2^ (Fig. 1f), suggesting an expansion of the electrochemically accessible surface area.

Next, we compared the fully activated electrode (FePc/Ni-25CV) with the pristine electrode (FePc/Ni-0CV) and bare Ni plate to highlight the performance gain enabled by reconstruction. Consistent with the rapid performance enhancement observed during the first 25 CV cycles, FePc/Ni-25CV required an overpotential that was 28 mV and 51 mV lower than those of FePc/Ni-0CV and the bare Ni plate, respectively, to achieve a current density of 10 mA cm^−2^ (Fig. 1g). Concurrently, the Tafel slope decreased to 42 mV dec^−1^, outperforming both the fresh catalyst (65 mV dec^−1^) and bare Ni (104 mV dec^−1^) (Fig. 1h), indicating a more favourable OER pathway on the reconstructed surface. Consistently, FePc/Ni-25CV exhibited the smallest *R*_ct_ (0.4 Ω), compared with FePc/Ni-0CV (0.6 Ω) and bare Ni (1.9 Ω) (Fig. 1i), underscoring the formation of a highly conductive interface that facilitates interfacial charge transfer and thereby accelerates OER kinetics. To examine whether this current enhancement could arise from Pc ligand-related oxidation, metal-free phthalocyanine on Ni (H_2_Pc/Ni) was examined after the same 25-cycle CV activation protocol. Its much lower current response and the nearly unchanged FePc/Ni-25CV polarization curve after subtracting the H_2_Pc/Ni-25CV current further supported that the enhanced OER activity primarily originated from the reconstructed Fe-containing interface on Ni rather than Pc ligand-related oxidation (Fig. S7). Together, these results indicated that the 25-cycle CV protocol drove a dynamic yet self-limiting reconstruction process, wherein FePc and the Ni substrate cooperatively evolved into a stable, high-activity bimetallic interface that accounted for the enhanced OER performance. FePc/Ni-25CV further sustained 100 h of chronopotentiometry at 100 mA cm^−2^ in 1 M KOH, with a potential increase of 0.12 V (Fig. S8). SEM comparison before and after the stability test showed no obvious morphological changes in representative regions (Fig. S9). Furthermore, a literature comparison based on geometric activity metrics and apparent TOF values showed that FePc/Ni-25CV exhibited a competitive apparent TOF, reaching 0.331 s^−1^ at *η* = 300 mV (Table S3).

### Structural amorphization and electronic evolution upon reconstruction

To identify the true active phase responsible for the enhanced OER performance, comprehensive structural analysis was carried out on the FePc/Ni electrode after 25-cycle CV activation. Transmission electron microscopy (TEM) revealed a uniform, featureless surface layer devoid of distinct lattice fringes (Fig. S10), suggesting an amorphous layer on the activated surface. In contrast, pristine FePc powder exhibited a well-defined lamellar morphology (Fig. S11) and sharp X-ray diffraction (XRD) peaks at 2*θ* values of 7.1°, 9.3°, and 27.3° (Fig. S12), matching crystalline FePc (PDF#14-0926), confirming its initial long-range order.^38^ The XRD pattern of the activated FePc/Ni-25CV electrode was dominated by the reflections from the metallic Ni substrate (Fig. S13). This is reasonable considering the low mass and poor crystallinity of the near-surface reconstructed thin layer. Together with the HRTEM observation of a featureless surface and the subsequently discussed XAS evidence for disrupted Fe–N_4_ coordination, these results supported the substantial transformation of the molecular precursor after the activation process. XPS-based surface composition analysis further confirmed the near-surface coexistence of Fe, Ni, and O in the activated FePc/Ni sample, with the corresponding atomic ratio summarized in Table S4. This result is consistent with the formation of an Fe/Ni-containing reconstructed interfacial region after electrochemical activation. High-resolution XPS spectra were further collected to probe the surface chemical states of FePc/Ni-25CV after activation. The Fe 2p spectrum (Fig. S14) exhibited characteristic Fe^3+^ features, with the Fe 2p_3/2_ peak located at ∼711.2 eV, while the O 1s spectrum (Fig. S15) contained metal–oxygen and hydroxyl/oxyhydroxide-related components at ∼529.6 and ∼531.7 eV, respectively. In addition, the Ni 2p spectrum (Fig. S16) was dominated by oxidized Ni species, suggesting near-surface oxidation of the Ni substrate during CV activation. These surface-sensitive XPS results indicate that CV activation generated an oxidized reconstructed surface environment involving Fe and Ni species. Collectively, these observations indicated that electrochemical activation reconstructed the FePc/Ni interface. This reconstructed interfacial layer, rather than the as-deposited molecular film, constituted the catalytically active entity that underpinned the observed OER enhancement.

**Fig. 2:**
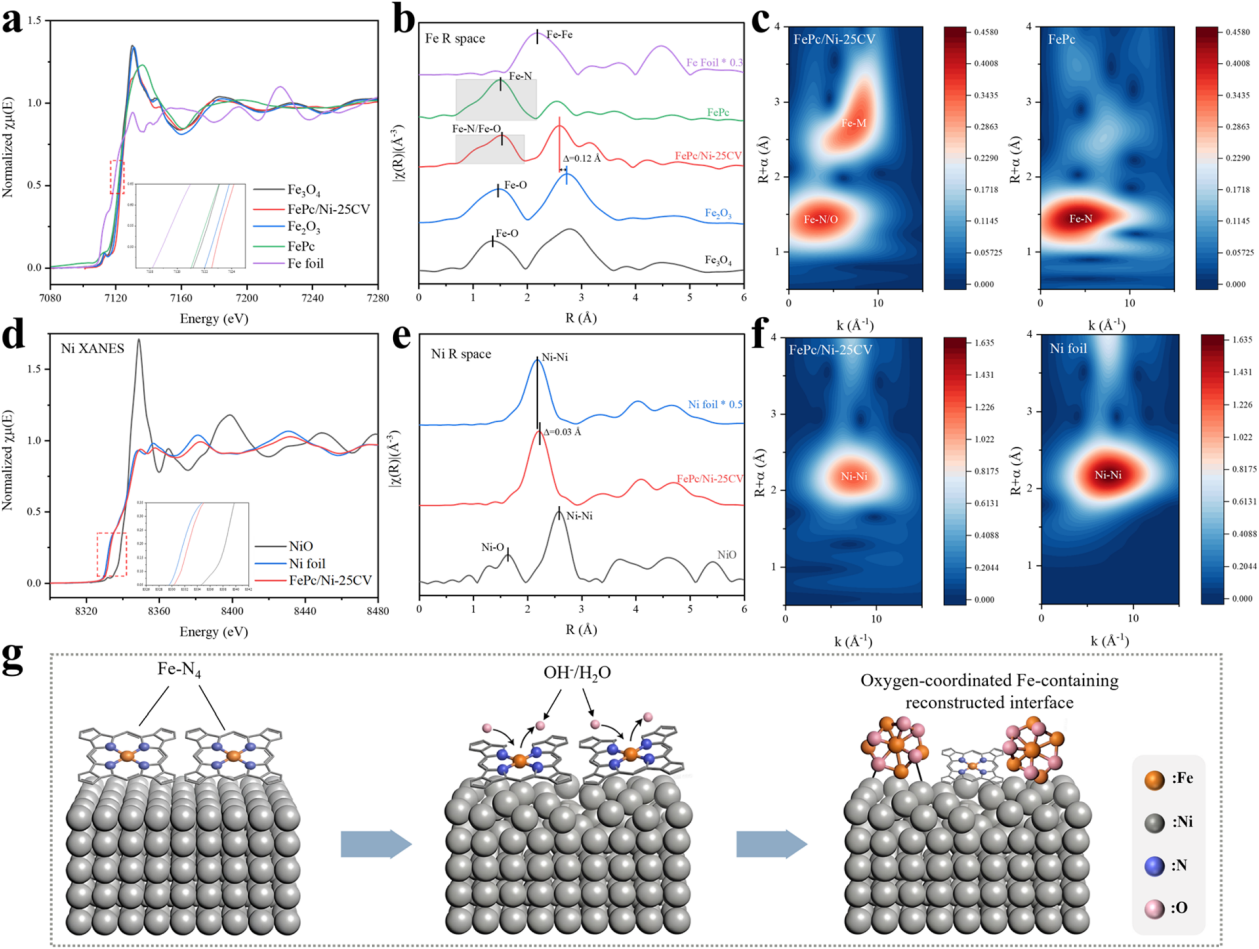
XANES and EXAFS characterization of FePc/Ni-25CV and reference samples. (a) Fe K-edge XANES spectra of FePc/Ni-25CV. (b) Fourier-transformed EXAFS spectra of FePc/Ni-25CV and references at the Fe K-edge. (c) Wavelet transform plots of Fe K-edge EXAFS signals for FePc/Ni-25CV and FePc. (d) Ni K-edge XANES spectra of FePc/Ni-25CV. (e) Fourier-transformed EXAFS spectra of FePc/Ni-25CV and references at the Ni K-edge. (f) Wavelet transform plots of Ni K-edge EXAFS signals for FePc/Ni-25CV and Ni foil. (g) Schematic illustration of XAS-inferred interfacial structural evolution at the FePc/Ni interface.

Synchrotron-based X-ray absorption spectroscopy (XAS) was utilized to probe the electronic and local structural evolution of Fe and Ni at the FePc/Ni interface after 25-cycle CV activation. X-ray absorption near-edge structure (XANES) and extended X-ray absorption fine structure (EXAFS) measurements offered insight into the interfacial interactions between FePc and the Ni substrate. The Fe K-edge XANES of the activated electrode showed a weaker pre-edge feature than pristine FePc (Fig. S17), consistent with modification of the original molecular Fe–N_4_ coordination environment after activation. In addition, the Fe K-edge position of FePc/Ni-25CV shifted to higher energy relative to pristine FePc and was located at slightly higher energy than that of Fe_2_O_3_, as shown by the XANES spectra and the quantitative edge-position analysis (Fig. 2a and Table S5), supporting a more oxidized Fe environment during activation. Linear combination fitting of the Fe K-edge XANES spectrum using FePc and Fe_2_O_3_ as references further revealed that FePc/Ni-25CV was dominated by an Fe_2_O_3_-like spectral contribution, with only a minor FePc-like component remaining (Fig. S18 and Table S6). This result supports the substantial loss of the original Fe–N_4_ coordination environment in FePc and the predominance of Fe–O coordination features in the reconstructed interface.

This transformation was corroborated by Fe K-edge EXAFS (Fig. 2b). The first-shell feature at around 1.5 Å without phase correction, which is characteristic of molecular Fe–N coordination in pristine FePc, is weakened after activation, consistent with the modification of the original molecular Fe–N coordination environment. Notably, FePc/Ni-25CV exhibited a higher-R feature at approximately 2.61 Å, which differed from the corresponding feature of bulk Fe_2_O_3_ at approximately 2.73 Å. This difference did not support the formation of bulk iron oxide. Rather, together with the weakened Fe–N feature and the positive Fe K-edge shift, these results were consistent with the formation of a more oxidized, oxygen-coordinated Fe-containing reconstructed interfacial environment, possibly involving Fe–O–M motifs (M = Fe and/or Ni). Wavelet transform analysis at the Fe K-edge (Fig. 2c) further supported these results. Pristine FePc exhibited a single intensity maximum in the low R/k region, whereas bulk Fe_2_O_3_ showed two characteristic features corresponding to the Fe–O contribution at low R/k and a metal-neighbour contribution at higher R/k (Fig. S19). Upon activation, FePc/Ni-25CV showed a modified low-R feature, while a high-R/high-k intensity emerged, further indicating disruption of the original molecular Fe environment and the appearance of higher-shell metal-neighbour scattering contributions.

In parallel, the Ni K-edge XANES spectrum of FePc/Ni-25CV exhibited only a minor positive edge shift relative to metallic Ni foil (Fig. 2d and Table S7), indicating that the bulk substrate remained largely metallic with only limited oxidation. However, the corresponding EXAFS showed noticeable broadening of the main Ni–Ni peak at ∼2.2 Å relative to Ni foil (Fig. 2e), reflecting increased local structural disorder around Ni. The wavelet transform of the Ni K-edge (Fig. 2f) showed that the Ni–Ni feature in FePc/Ni-25CV remained in a similar R/k region to Ni foil but became diffuse and markedly weakened. Meanwhile, the characteristic Ni–O intensity peak observed in the low R/low k region for the NiO reference sample (Fig. S20) was absent in FePc/Ni-25CV, making the formation of bulk NiO unlikely. Together, these data suggested that the Ni atoms underwent subtle oxidation and structural reconstruction without compromising the conductive metallic nature of the current collector.

Collectively, the XAS results indicated interfacial reconstruction in which molecular FePc evolved into more oxidized Fe-containing environments with oxygen coordination and an amorphous local structure (Fig. 2g). These Fe centres were associated with the Ni surface environment, possibly through oxygen-mediated Fe–O–M motifs (M = Fe and/or Ni) at the interface. Such a reconstructed Fe/Ni-containing interfacial environment, rather than the initial molecular film or bulk oxide phases, was therefore considered the catalytically relevant active state responsible for the enhanced OER kinetics. This hybrid interface integrated oxidized Fe components with the conductive and dynamically evolving Ni-based scaffold.

### Dynamic reconstruction and interfacial kinetics for enhanced OER activity

To establish a direct connection between interfacial reconstruction and OER enhancement, we correlated real-time structural evolution with adsorption and reaction kinetics during CV activation. Based on the post-reconstruction characterization of morphology, electronic structure, and coordination environment, we employed *in situ* electrochemical quartz-crystal microbalance with dissipation (EQCM-D) to track the mass and viscoelastic changes at the FePc/Ni interface across successive CV cycles.

**Fig. 3:**
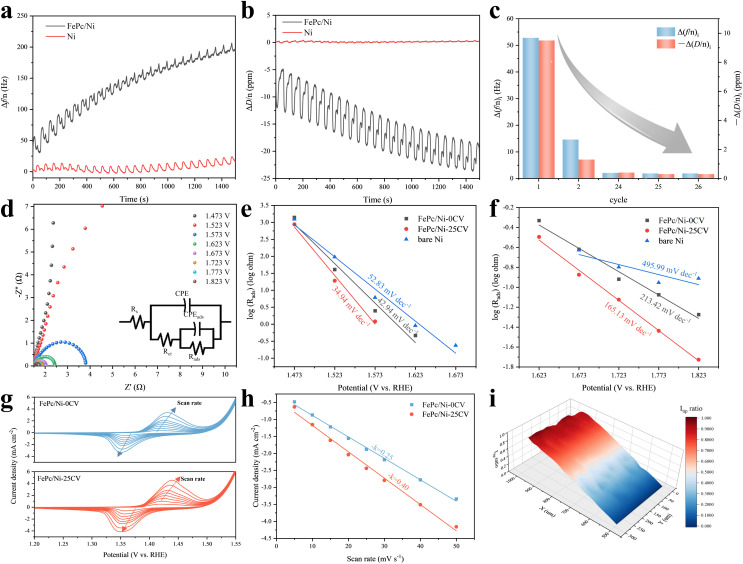
Mechanism of interfacial evolution and OER activity enhancement. (a) Δ*f*/*n vs.* time during CV activation for FePc/Ni and bare Ni. (b) Δ*D*/*n vs.* time during CV activation for FePc/Ni and bare Ni. (c) Net per-cycle EQCM-D shifts for selected cycles (*i* = 1, 2, 24, 25, 26). For each cycle, the normalized frequency and dissipation changes were calculated by subtracting the start-of-cycle values from the corresponding end-of-cycle values; dissipation changes are plotted with an inverted sign for clarity. (d) Potential-dependent EIS spectra collected at different applied potentials. (e and f) Potential-dependent adsorption resistance (*R*_ads_) extracted from potential-dependent EIS by equivalent-circuit fitting. (g) CV curves of FePc/Ni-0CV and FePc/Ni-25CV at different scan rates. (h) Linear fitting of the cathodic peak current density *versus* scan rate. (i) SECM activity maps of FePc/Ni-25CV and Ni.

*Operando* EQCM-D traces recorded at the third overtone showed that FePc/Ni underwent significant interfacial evolution during CV activation, in stark contrast to bare Ni, which exhibited only minor changes (Fig. 3a and b). The traces for FePc/Ni showed pronounced baseline drift in both Δ*f*/*n* and Δ*D*/*n*, superimposed with oscillations synchronized to each CV cycle. The intra-cycle oscillations reflected reversible, potential-driven responses, while the multi-cycle drift signified cumulative, irreversible reconstruction. Notably, Δ*D*/*n* became progressively more negative with cycling, suggesting transformation of the initial organic FePc layer into a denser and stiffer inorganic-like interface. This irreversible evolution was most intense in the early cycles and gradually diminished as activation proceeded (Fig. 3c). Quantitatively, the first cycle yielded a net Δ*f*/*n* increase of +52.8 Hz (Fig. S21) accompanied by a net Δ*D*/*n* change of −9.5 ppm (Fig. S22), indicative of substantial mass loss coupled with interfacial stiffening. By cycle 2, these net shifts diminished markedly to +14.6 Hz (Fig. S23) and −1.3 ppm (Fig. S24). The trend continued, with net changes of +2.1 Hz (Fig. S25) and −0.4 ppm (Fig. S26) in cycle 24. Notably, the changes in cycle 25 (+1.9 Hz and −0.3 ppm, Fig. S27 and S28) were identical to those in cycle 26 (+1.9 Hz and −0.3 ppm, Fig. S29 and S30), indicating that the irreversible reconstruction process was effectively complete by this stage. The residual EQCM-D response beyond cycle 25 was thus dominated by reversible interfacial processes, suggesting the establishment of a structurally stable active interface, consistent with the CV and polarization curves.

To further probe the evolution of surface-active sites during CV activation, we analysed the third-harmonic Fourier-transformed alternating current voltammetry (FTacV) response of FePc/Ni in the potential range of 1.35–1.45 V *vs.* RHE, which displayed two main peaks (Fig. S31). As the number of cycles increased from 0 to 5, 10, and 25, both peaks shifted slightly towards more positive potentials, with the intensity of the low-potential peak continuously increasing, while the intensity of the high-potential peak gradually decreased. This likely reflected the modulation and progressive evolution of the corresponding electrochemically active interfacial sites, consistent with the gradual formation of a reconstructed interface during CV activation.

To gain deeper insight into the charge transfer kinetics during the OER and the effect of electrochemical reconstruction on the interfacial adsorption behaviour of reaction intermediates, potential-dependent EIS measurements were performed. In the Nyquist plot of FePc/Ni-25CV, the semicircle gradually shrank with increasing potential, indicating that the interfacial charge transfer accelerated with potential (Fig. 3d). A similar phenomenon was observed for the original FePc/Ni and pure Ni (Fig. S32 and S33). Furthermore, an equivalent circuit incorporating the adsorption process was employed to fit the EIS data, and the extracted reactive OER intermediate adsorption resistance (*R*_ads_) was used for analysis.^39–41^ The potential dependence of log(*R*_ads_) was then compared for fresh FePc/Ni, FePc/Ni-25CV and bare Ni (Fig. S34). The results showed that, over the entire potential window, the FePc/Ni-25CV electrode exhibited significantly lower *R*_ads_ values compared to the other two electrodes, indicating that intermediate adsorption was notably facilitated on the reconstructed FePc/Ni interface. Further kinetic analysis (Fig. 3e and f) showed that, in two distinct potential regions, log(*R*_ads_) varied linearly with potential, with FePc/Ni-25CV exhibiting a markedly steeper apparent slope than the other electrodes. These results indicated that CV activation not only lowered the absolute resistance associated with intermediate adsorption on FePc/Ni, but also enhanced the adsorption kinetics, thereby providing kinetic evidence for the improved OER activity of the reconstructed interface.

To further investigate the dynamic adsorption characteristics of the reconstructed interface, CV was performed at different scan rates within the characteristic redox potential window (Fig. 3g). Clear redox peaks were observed for both the fresh and the activated FePc/Ni electrodes, which could be attributed to the redox processes of the relevant active metals. The reduction peak current density (*j*_p_) exhibited a linear dependence on the scan rate (*v*), with the slope being directly proportional to the surface coverage (*Γ*) of the electroactive species involved in the reaction (Fig. 3h). The linear fitting slope for FePc/Ni-25CV was significantly larger than that for the initial FePc/Ni, indicating a marked increase in the coverage of redox-active oxygen-containing species on the reconstructed interface. Combining the previously discussed potential-dependent EIS results showing a significant decrease in *R*_ads_, it was reasonable to propose that the electrochemical reconstruction constructed a high-density and highly active interface on FePc/Ni. This reconstructed interface directly accelerated the adsorption of intermediates, thereby providing a kinetic basis for the enhanced OER performance.

The spatial uniformity and local catalytic activity of this reconstructed interface were mapped by scanning electrochemical microscopy (SECM) in substrate generation/tip collection (SG/TC) mode (Fig. 3i). The FePc modified region exhibited a higher tip current (red region), consistent with enhanced local OER activity, whereas the bare Ni area showed minimal activity (blue region). This microscale mapping supported the conclusion that the enhanced OER performance originated from a catalytically uniform interface formed through reconstruction. Collectively, the CV activation process converted FePc/Ni into a continuous and highly active interfacial state, featuring facilitated intermediate adsorption and charge transfer, thereby accounting for the enhanced OER performance.

### Decoupling the roles of the metal centre and substrate in reconstructed OER interfaces

**Fig. 4:**
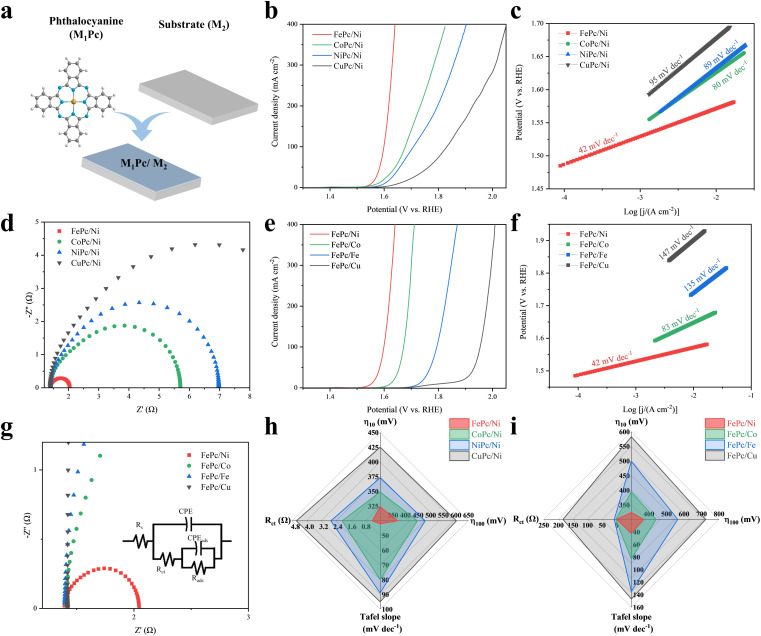
OER performance of M_1_Pc/M_2_ after CV activation. (a) Schematic diagram of different metal phthalocyanines loaded on different metal substrates. (b and e) LSV curves, (c and f) Tafel plots and (d and g) EIS spectra of M_1_Pc/M_2_. (h and i) Radar plots of M_1_Pc/M_2_ for OER performance.

Having established the structural characteristics and kinetic origins of enhanced OER activity in the FePc/Ni system, we next sought to decouple the individual contributions of M_1_ and M_2_. To this end, we constructed a combinatorial library of M_1_Pc/M_2_ electrodes (M_1_ and M_2_ = Fe, Co, Ni, Cu) and evaluated their OER performance after the same standardized 25-cycle CV activation protocol used for FePc/Ni-25CV (Fig. 4a). First, with Ni as the fixed substrate, varying M_1_ yielded a clear activity trend of FePc/Ni > CoPc/Ni > NiPc/Ni > CuPc/Ni (Fig. 4b and c). In detail, CuPc/Ni required an overpotential of 425 mV to reach 10 mA cm^−2^, 101 mV higher than that of FePc/Ni, and displayed sluggish kinetics with a high Tafel slope of 95 mV dec^−1^. EIS further corroborated a significantly lower *R*_ct_ for FePc/Ni *versus* CuPc/Ni (Fig. 4d), reflecting faster interfacial electron transfer. The as-deposited M_1_Pc/Ni films were further characterized by SEM to compare their initial deposition morphologies (Fig. S35). Although distinct differences in surface coverage and aggregate morphology were observed among the samples, these variations did not show a simple correspondence with the OER activity trend. This observation suggested that the M_1_Pc/Ni-dependent activity trend was unlikely to originate solely from differences in the pristine spray-coated morphology. Crucially, the pre-OER anodic features in the initial CV scans suggested that M_1_ modulated the electrochemical reconstruction of the Ni substrate, with the bare Ni exhibiting a broad oxidation peak at ∼1.404 V *vs.* RHE while the M_1_Pc/Ni electrodes shifted this feature positively to ∼1.441 V (CoPc/Ni), ∼1.446 V (NiPc/Ni) and ∼1.497 V (CuPc/Ni), accompanied by altered peak shapes (Fig. S36). These signatures suggested that M_1_ influenced the anodic oxidation behaviour of Ni during activation, potentially by modifying the interfacial charge distribution or acting as a redox mediator, thereby affecting the formation of the reconstructed interface.

Conversely, with M_1_ fixed as Fe, varying M_2_ produced the trend of FePc/Ni > FePc/Co > FePc/Fe > FePc/Cu (Fig. 4e and f), consistent with the corresponding EIS-derived charge-transfer kinetics (Fig. 4g). This indicated that M_2_ was not merely a conductive support but an active host that influenced the formation of the catalytic interface. Ni and Co substrates served as ideal “host frameworks” because their surface (oxy)hydroxides were intrinsically active for the OER in alkaline media and could support efficient interfacial electron transport. In contrast, Cu-based surfaces exhibited relatively low intrinsic OER activity, limiting any synergistic benefit from Fe incorporation. FePc deposited on carbon paper (FePc/CP) was further examined after the same 25-cycle CV activation protocol, but it showed no appreciable pre-OER redox response (Fig. S37) and required a much higher overpotential of 542 mV to reach 10 mA cm^−2^ (Fig. S38). This result further highlighted the essential role of the Ni host in forming the active reconstructed interface.

To rigorously test the proposed roles of M_1_ and M_2_, we compared FePc/Ni with its elemental swap counterpart of NiPc/Fe. Despite containing identical elements, NiPc/Fe exhibited markedly inferior performance with overpotentials of 483 mV and 544 mV at 10 and 100 mA cm^−2^, respectively, which were more than 150 mV higher than those of FePc/Ni (Fig. S39). In addition, NiPc/Fe exhibited a large Tafel slope of 64 mV dec^−1^ (Fig. S40), high *R*_ct_ of 3.77 Ω and high *R*_ads_ of 204 Ω, as well as a small *C*_dl_ of 0.08 mF cm^−2^ (Fig. S41 and S42), indicating that the reconstruction effectiveness and interfacial quality were highly sensitive to which element served as M_1_*versus* M_2_. Replacing FePc with NiPc changed the molecular metal centre, thereby altering its redox characteristics and likely leading to a different reconstruction behaviour during activation, while using Fe as the substrate may weaken the ability of the host to serve as a conductive and intrinsically active (oxy)hydroxide framework.^42,43^ This interpretation is also supported by the different CV evolution behaviour of FePc/Ni and NiPc/Fe during activation (Fig. S43 and S44). Compared with FePc/Ni, NiPc/Fe showed a weaker activation response and much lower anodic current, further highlighting that the roles of the molecular centre and the metallic substrate are not simply interchangeable in directing interfacial reconstruction.

Collectively, these bivariate experiments revealed two independent yet complementary design principles for high-performance reconstructed interfaces. On the molecular side, selecting the M_1_ centre with appropriate redox properties appeared beneficial for facilitating interfacial reconstruction and modulating the electronic environment. On the substrate side, choosing an M_2_ host capable of developing an efficient (oxy)hydroxide framework helped support cooperative interaction with M_1_. The optimal OER performance was typically achieved by satisfying both criteria *via* an appropriate pairing of the M_1_ centre with M_2_, as exemplified by FePc/Ni. The comprehensive performance landscape, summarized in radar plots of *η*_10_ (324 to 583 mV), the Tafel slope (42 to 147 mV dec^−1^), and *R*_ct_ (0.44 to 184 Ω, spanning >2 orders of magnitude) (Fig. 4h, i and Table S8), strongly supported this dual-parameter model and provided a predictive framework for identifying high-performance M_1_–M_2_ combinations beyond this study.

## Conclusion

In summary, this work demonstrated that electrochemical activation transformed molecular FePc on Ni into a highly active, near-surface, oxygen-coordinated Fe-containing reconstructed interface. This interface facilitates the adsorption of reaction intermediates and charge transfer for enhanced OER. The FePc/Ni model system thus provides mechanistic insight into how a molecular precursor and a metallic substrate cooperatively evolve during activation to generate a catalytically competent reconstructed interface. The comparative M_1_Pc/M_2_ study further showed that this mechanistic understanding can be extended into a broader design strategy for reconstructed OER interfaces. By systematically varying the phthalocyanine metal centre (M_1_) and substrate metal (M_2_), we established the key design principle that M_1_ should possess appropriate redox properties to facilitate interfacial reconstruction and M_2_ could serve as an active host. The synergy between these roles was exemplified by FePc/Ni, whereas the poor performance of swapped systems like NiPc/Fe supported their non-interchangeability. These insights establish a reconstruction–hybridization strategy in which suitable M_1_Pc precursors are paired with active M_2_ substrates to enable the *in situ* formation of catalytically effective reconstructed OER interfaces, providing a rational pathway for catalyst design beyond this study.

## Author contributions

X. L. conceived the concept, directed the project, and reviewed and edited the manuscript. X. F. Q. supervised the project and reviewed the manuscript. F. W. and D. L. Z. carried out the experiments and wrote the initial draft of the manuscript. Y. B. Z. conducted the investigation and carried out the data analysis. H. J. L. and G. Q. S. performed the synchrotron experiments and conducted the data analysis. P. L. assisted with data collection and analysis. All authors have approved the final version of the manuscript.

## Conflicts of interest

There are no conflicts to declare.

## Supplementary Material

SC-OLF-D6SC02174C-s001

## Data Availability

The data supporting this article have been included as part of the supplementary information (SI). Supplementary information: experimental details, additional characterization results, electrochemical analyses, and supporting figures and tables. See DOI: https://doi.org/10.1039/d6sc02174c.

## References

[cit1] Colelli F. P., Johannes E., Giacomo M., Mistry M. N., De Cian E. (2022). Nat. Commun..

[cit2] Seh Z. W., Kibsgaard J., Dickens C. F., Chorkendorff I., Nørskov J. K., Jaramillo T. F. (2017). Science.

[cit3] Liu R.-T., Xu Z.-L., Li F.-M., Chen F.-Y., Yu J.-Y., Yan Y., Chen Y., Xia B. Y. (2023). Chem. Soc. Rev..

[cit4] Lei J., Wang Z., Zhang Y., Ju M., Fei H., Wang S., Fu C., Yuan X., Fu Q., Farid M. U., Kong H., An A. K., Deng R., Liu F., Wang J. (2024). Carbon Neutrality.

[cit5] Slobodkin I., Davydova E., Sananis M., Breytus A., Rothschild A. (2024). Nat. Mater..

[cit6] Sun H., Xu X., Kim H., Jung W., Zhou W., Shao Z. (2023). Energy Environ. Mater..

[cit7] Grigoriev S. A., Fateev V. N., Bessarabov D. G., Millet P. (2020). Int. J. Hydrogen Energy.

[cit8] Qian Q., Zhu Y., Ahmad N., Feng Y., Zhang H., Cheng M., Liu H., Xiao C., Zhang G., Xie Y. (2024). Adv. Mater..

[cit9] Li H., Lin Y., Duan J., Wen Q., Liu Y., Zhai T. (2024). Chem. Soc. Rev..

[cit10] Kang W., Wei R., Yin H., Li D., Chen Z., Huang Q., Zhang P., Jing H., Wang X., Li C. (2023). J. Am. Chem. Soc..

[cit11] Zhou Y., Wang Z., Cui M., Wu H., Liu Y., Ou Q., Tian X., Zhang S. (2024). Adv. Funct. Mater..

[cit12] Yu M., Budiyanto E., Tüysüz H. (2022). Angew. Chem., Int. Ed..

[cit13] Gaikwad M. A., Burungale V. V., Malavekar D. B., Ghorpade U. V., Suryawanshi U. P., Jang S., Guo X., Shin S. W., Ha J.-S., Suryawanshi M. P., Kim J. H. (2024). Adv. Energy Mater..

[cit14] Hao Y., Li Y., Wu J., Meng L., Wang J., Jia C., Liu T., Yang X., Liu Z.-P., Gong M. (2021). J. Am. Chem. Soc..

[cit15] Luo X., Zhao H., Tan X., Lin S., Yu K., Mu X., Tao Z., Ji P., Mu S. (2024). Nat. Commun..

[cit16] Chen Y., Li Q., Lin Y., Liu J., Pan J., Hu J., Xu X. (2024). Nat. Commun..

[cit17] Qu J., Dong Y., Zhang T., Zhao C., Wei L., Guan X. (2024). Front. Energy.

[cit18] Li X., Wang J., Xue H., Zhao L., Lu J., Zhang H., Yan M., Deng F., Hu C. (2025). Adv. Funct. Mater..

[cit19] Halldin Stenlid J., Görlin M., Diaz-Morales O., Davies B., Grigorev V., Degerman D., Kalinko A., Börner M., Shipilin M., Bauer M., Gallo A., Abild-Pedersen F., Bajdich M., Nilsson A., Koroidov S. (2025). J. Am. Chem. Soc..

[cit20] Wang X., Peng Z., Zhou W., Chen X., Tan Y., Huang Y.-F., Liu Z., Deng W.-Q., Wu H. (2025). Angew. Chem., Int. Ed..

[cit21] Zhou D., Li P., Lin X., McKinley A., Kuang Y., Liu W., Lin W.-F., Sun X., Duan X. (2021). Chem. Soc. Rev..

[cit22] Yu H., Sweers M. E., Osmieri L., Park J. H., Kropf A. J., Yang D., Ma L., Lyu X., Serov A., Cullen D. A., Zelenay P., Myers D. J., Hermann R. P. (2025). EES Catal..

[cit23] Li Z., Zhou Z., Sun M., Wu T., Lu Q., Lu L., Chen B., Chan C. H., Wong H. H., Huang B. (2025). Chem. Sci..

[cit24] Kumar A., Kumar Vashistha V., Kumar Das D. (2021). Coord. Chem. Rev..

[cit25] Yang S., Yu Y., Gao X., Zhang Z., Wang F. (2021). Chem. Soc. Rev..

[cit26] Zhang J., My Pham T. H., Gao Z., Li M., Ko Y., Lombardo L., Zhao W., Luo W., Züttel A. (2023). ACS Catal..

[cit27] Qi Q., Guan W., Zi Y., Zhang C., Hu J. (2026). Adv. Funct. Mater..

[cit28] Chen H., Wang L., Na M., Zou X. (2025). Chem. Sci..

[cit29] Zhang Z., Zhao H., Xi S., Zhao X., Chi X., Bin Yang H., Chen Z., Yu X., Wang Y.-G., Liu B., Chen P. (2025). Nat. Commun..

[cit30] Fang C., Zhou J., Zhang L., Wan W., Ding Y., Sun X. (2023). Nat. Commun..

[cit31] Bai L., Hsu C.-S., Alexander D. T. L., Chen H. M., Hu X. (2021). Nat. Energy.

[cit32] Ou Y., Twight L.
P., Samanta B., Liu L., Biswas S., Fehrs J. L., Sagui N. A., Villalobos J., Morales-Santelices J., Antipin D., Risch M., Toroker M. C., Boettcher S. W. (2023). Nat. Commun..

[cit33] He Z., Zhang J., Gong Z., Lei H., Zhou D., Zhang N., Mai W., Zhao S., Chen Y. (2022). Nat. Commun..

[cit34] Wu D., Hu L., Liu X., Liu T., Zhu X., Luo Q., Zhang H., Cao L., Yang J., Jiang Z., Yao T. (2025). Nat. Commun..

[cit35] Jiang Q., Wang S., Zhang C., Sheng Z., Zhang H., Feng R., Ni Y., Tang X., Gu Y., Zhou X., Lee S., Zhang D., Song F. (2023). Nat. Commun..

[cit36] Zhu M., Luo H., Bao H., Pan Z., Zhang S., Li Z., Zhang D. (2026). ACS Appl. Mater. Interfaces.

[cit37] Xu S., Ding Y., Du J., Zhu Y., Liu G., Wen Z., Liu X., Shi Y., Gao H., Sun L., Li F. (2022). ACS Catal..

[cit38] Zhou S., Sun H., Wang J., Cui H., Wang J. (2025). J. Alloys Compd..

[cit39] Ying M., Tang R., Zhao S., Yang W., Liang W., Zhang X., Yang G., Zheng R., Pan H., Liao X., Huang J. (2021). Adv. Energy Sustainability Res..

[cit40] Wang C., Deng C., Zhai P., Shi X., Liu W., Jin D., Shang B., Gao J., Sun L., Hou J. (2025). Nat. Commun..

[cit41] Jeon S. S., Kang P. W., Klingenhof M., Lee H., Dionigi F., Strasser P. (2023). ACS Catal..

[cit42] Yang S., Lu L., Zhan P., Si Z., Chen L., Zhuang Y., Qin P. (2024). Appl. Catal., B.

[cit43] Chen S., Su F., Gao Y., Li Z., Li H. (2025). ACS Appl. Nano Mater..

