# Supplementary material for: Reconstruction–hybridization of molecular and metallic interfaces for efficient oxygen evolution
Source: Chem Sci. 2026 May 27;17(27):13562–71. doi: 10.1039/d6sc02174c (PMC13231903; doi:10.1039/d6sc02174c)
Supplement: SC-017-D6SC02174C-s001 [file SC-017-D6SC02174C-s001.pdf]

Supplementary Information (SI) for Chemical Science.
This journal is © The Royal Society of Chemistry 2026

# 1 Experimental Section

## 1.1 Materials

Iron phthalocyanine (FePc, 92%) was purchased from Shanghai yuanye Bio-Technology Co., Ltd. Cobalt phthalocyanine (CoPc, 95%), nickel phthalocyanine (NiPc, > 97%) and Nafion perfluorinated resin aqueous dispersion solution were bought from Shanghai Titan Scientific Co., Ltd. Copper phthalocyanine (CuPc, 90%) was purchased from Shanghai Macklin Biochemical Co., Ltd. Ethanol ($C_2H_5OH$) were purchased from Sinopharm Chemical Reagent Co. Ltd. Ultrapure deionized water (18.2 MΩ cm) was used in all experiments. All chemicals were used without further purification.

## 1.2 Characterization

The crystal structure of the original iron phthalocyanine was analysed by powder X-ray diffraction (XRD, Bruker D8 Advance) using Cu Kα radiation over a 2θ range of 5-80°. The morphology and microstructure of the original iron phthalocyanine were characterized using scanning electron microscopy (SEM) (ZEISS SUPRA ®55). Transmission electron microscopy (TEM) of the activated electrode material (after 25 CV cycles) was performed on a JEOL JEM-2100F microscope. Micro-attenuated total reflection Fourier transform infrared (micro-ATR-FTIR) spectroscopy was performed on a Thermo Scientific Nicolet iN10 MX FTIR microscope to acquire near-surface vibrational fingerprints of the electrodes. Spectra were collected with a spectral resolution of 4 $cm^{-1}$ and 32 scans.

X-ray absorption spectroscopy (XAS) experiments were performed at the Fe and Ni K edges at the MEX-1 beamline of the Australian Synchrotron. Both X-ray absorption near-edge structure (XANES) and extended X-ray absorption fine structure (EXAFS) spectra were collected. The photon-energy calibration was referenced to the corresponding metal foils by aligning the first-derivative maximum (first inflection point) of the foil spectra. The obtained EXAFS data were processed and fitted using Athena and Artemis software to analyse the local coordination environment and electronic structure of metal centres.[1]

## 1.3 Electrochemical measurement

Electrochemical measurements were conducted on a CHI 760E electrochemical workstation (CH Instruments Inc.) using a standard three-electrode system. A platinum sheet was used as the counter electrode, and an Ag/AgCl (saturated KCl) electrode was employed as the reference electrode.

To prepare the catalyst ink, 6 mg of metal phthalocyanine (MPc) powder was dispersed in 6 mL of ethanol, followed by the addition of 6 μL of a Nafion perfluorinated resin aqueous dispersion solution. The mixture was ultrasonicated to obtain a homogeneous ink. The working electrode was fabricated by spray-coating the as-prepared ink onto a pre-cleaned metal foil substrate (1 × 2 cm), with the catalyst-coated active area defined as 1 × 1 cm. The ink volume was adjusted to achieve the targeted catalyst loading corresponding to the 1 $cm^2$ active area. The electrode was dried under ambient conditions before electrochemical measurements.

All electrochemical measurements were performed in 1 M KOH at room temperature. All potentials measured against the Ag/AgCl reference electrode were calibrated to the reversible hydrogen electrode (RHE) according to the Nernst equation: E(vs. RHE) = E(vs. Ag/AgCl) + 0.197 V + pH × 0.059 V. And the OER overpotential ($\eta$) was calculated as: $\eta$ = E(vs. RHE) – 1.23 V.

Electrochemical activation of FePc/Ni electrodes was conducted using three protocols, including cyclic voltammetry (CV) cycling, potentiostatic activation (chronoamperometry, CA) at fixed potentials, and a multi-potential potentiostatic step program (CA-STEP). The total activation time was kept identical for all protocols. CV was conducted at a scan rate of 10 mV $s^{-1}$ within the potential window of 1.023-1.623 V vs. RHE (25CV). Potentiostatic holds were performed at 1.223, 1.423, and 1.623 V vs. RHE (CA 1.223 V, CA 1.423 V, and CA 1.623 V). The CA-STEP protocol employed a symmetric potential-step sequence (vs. RHE): 1.023 V (15 s), 1.223 V (15 s), 1.423 V (15 s), 1.623 V (30 s), followed by 1.423 V (15 s), 1.223 V (15 s), and 1.023 V (15 s).

Linear sweep voltammetry (LSV) curves were recorded at 5 mV $s^{-1}$ with 95% iR compensation. Tafel slopes were determined at a scan rate of 0.5 mV $s^{-1}$ and presented without iR compensation. The electrochemical double-layer capacitance ($C_{dl}$) was

estimated from CV curves measured in the non-Faradaic region at scan rates from 20 to 100 mV $s^{-1}$. Chronopotentiometry (CP) was carried out under a constant current density of 10 mA $cm^{-2}$ and 100 mA $cm^{-2}$ in 1 M KOH at room temperature. Electrochemical impedance spectroscopy (EIS) was performed at selected potentials with a frequency range from 100 kHz to 0.01 Hz. The physical quantities involved in EIS and their meanings are shown in the following table:

| Physical quantity | Unit | Meaning |
|---|---|---|
| $R_s$ | Ω | The resistance of the solution |
| *CPE* | F | The double-layer capacitance |
| $R_{ct}$ | Ω | Charge transfer resistance of surface reaction |
| $CPE_{ads}$ | F | The relaxation of the charge of oxygenated intermediates adsorption process |
| $R_{ads}$ | Ω | Charge transfer resistance of oxygenated intermediates adsorption process |

To probe the evolution of interfacial adsorption-related surface species after CV activation, cyclic voltammetry was recorded in a potential window covering the characteristic pre-OER redox features (1.2–1.55 V vs. RHE) at scan rates from 5 mV $s^{-1}$ to 50 mV $s^{-1}$. When the redox species are adsorbed on the electrode, the current density of the cathodic peak ($j_p$) can be linearly plotted against the scan rate (v), which can be expressed as:

$$j_p = \frac{n^2F^2}{4RT}v\Gamma$$

where n is the number of electrons transferred, F is the Faraday coefficient, Γ is the surface coverage of the adsorbed species.[2]

Fourier-transform alternating current voltammetry (FTacV) experiments were carried out in the same three-electrode system, using an applied sine wave with the amplitude of 60 mV and a frequency of 9.02 Hz. The alternating current (AC) signals could be transformed into direct current (DC) spectrum as well as the fundamental and

higher-order harmonic spectra (second, third, etc.). The higher harmonic components are very sensitive to the fast electron transfer processes and can avoid the interference of catalytic faradaic and background charging currents. The third harmonic spectrum was utilized for the data analysis.[3, 4]

**1.4 SECM characterization**

Scanning electrochemical microscopy (SECM) (CHI 920D, CH Instruments Inc.) measurement was performed to map the spatial distribution of OER activity. A Pt tip ($WE_{tip}$) with a diameter of 10 μm and RG value of 5 (CHI 116, CH Instruments Inc.) was used as the tip working electrode, with an Ag/AgCl electrode as the reference and a Pt wire as the counter electrode. The substrate working electrode ($WE_{sub}$) was a nickel plate with a FePc-coated region prepared by the same spray-coating procedure as that used for the working electrodes in the electrochemical tests, and this FePc-coated region was subsequently activated by 25 CV cycles and served as the working area, while the uncoated region of the nickel plate was used as the reference area for comparison. A sharp boundary between the two regions was created by covering the uncoated area with tape during spray-coating. The approach of the $WE_{tip}$ to the surface of the Ni plate was adjusted using a probe approach curve. Measurements were conducted in 1 M KOH under the substrate generation/tip collection (SG/TC) mode for *in situ* mapping of OER activity. During scanning, the potential of the Pt tip ($E_{tip}$) was held at 0.562 V vs. RHE and the potential of substrate ($E_{sub}$) was set to 1.623 V vs. RHE, with a scan step size of 10 μm for both the x and y directions. All SECM experiments were carried out in a Faraday cage at room temperature.

**1.5 *In situ* EQCM-D measurements**

Electrochemical quartz crystal microbalance with dissipation monitoring (EQCM-D) measurements were performed on a Q-Sense system (Biolin Scientific, Sweden). Au-coated quartz crystal sensors were used as the substrates for fabricating catalyst-coated working electrodes. A Pt sheet and an Ag/AgCl electrode were employed as the counter and reference electrodes, respectively, and 1 M KOH served as the electrolyte. Prior to measurements, the electrolyte was introduced into the system and the signals were allowed to stabilize, after which the baseline was set to zero. For the FePc/Ni

working electrode, Ni ink and FePc ink were sequentially spin-coated onto the Au sensor with drying after each coating, while a Ni-only electrode prepared identically but without the FePc layer was used for comparison. The EQCM-D responses were recorded operando during the CV activation protocol (25 cycles) using the same potential window as that used in the electrochemical tests.

**1.6 Determination of Fe and Ni K-edge edge positions**

The Fe and Ni K-edge positions of the reference compounds and FePc/Ni-25CV were extracted from the normalized XANES spectra using the integral method, following a previously reported procedure.[5, 6] In this approach, the edge position is defined from the main rising part of the absorption edge rather than from a single visual point, which reduces the influence of local spectral features and enables a more consistent comparison among different samples.

The average edge energy $E_{\mathrm{edge}}$ can be written as:

$$E_{\mathrm{edge}} = \frac{1}{\mu_1 - \mu_2} \int_{\mu_1}^{\mu_2} E(\mu)\, d\mu$$

where $\mu(E)$ is the normalized XANES spectrum and $E(\mu)$ is its inverse function.

The same quantity can also be expressed as:

$$E_{\mathrm{edge}} = E(\mu_1) + \frac{1}{\mu_1 - \mu_2} \int_{E(\mu_1)}^{E(\mu_2)} [\mu_2 - \mu(E)]\, dE$$

For experimental XANES data, however, $\mu(E)$ is discrete and may not be strictly monotonic, such that the inverse function is not uniquely defined over the entire edge region. Therefore, numerical integration was performed using a truncated absorption function, $\hat{\mu}(E)$, defined as:

$$\hat{\mu}(E) = \begin{cases} \mu_1, & \mu(E) < \mu_1 \\ \mu_2, & \mu(E) > \mu_2 \\ \mu(E), & \text{otherwise} \end{cases}$$

Accordingly, the edge position was calculated over a suitably chosen energy interval $[E_1, E_2]$ as:

$$E_{\text{edge}} = E_1 + \frac{1}{\mu_1 - \mu_2}\int_{E_1}^{E_2} \left[\mu_2 - \hat{\mu}(E)\right] dE$$

Here, $E_1$ and $E_2$ were chosen such that $\mu(E_1) < \mu_1$ and $\mu(E_2) > \mu_2$. Under these conditions, the calculated $E_{\text{edge}}$ is not sensitive to the specific choices of $E_1$ and $E_2$, whereas it depends on the selected values of $\mu_1$ and $\mu_2$. In this work, the $\mu_1$ and $\mu_2$ values for the Fe and Ni K-edge analyses were selected to represent the main rising-edge regions of the normalized XANES spectra, with $\mu_1$ = 0.2 and $\mu_2$ = 0.75 for Fe and $\mu_1$ = 0.05 and $\mu_2$ = 0.35 for Ni. These values were selected to represent the main rising-edge region while minimizing contributions from pre-edge features and white-line intensity.

**1.7 Estimation of ECSA-based TOF**

The ECSA-based turnover frequency (TOF) of FePc/Ni-25CV was estimated from the ECSA-normalized current density at an overpotential of 300 mV. The ECSA factor was calculated from the double-layer capacitance according to $C_{dl}/C_s$, where $C_s$ was taken as 40 μF $cm^{-2}$.

The TOF was calculated using:

$$TOF = \frac{j_{ECSA} N_A}{4F\Gamma}$$

where $N_A$ is Avogadro's constant, F is the Faraday constant, and the factor of 4 corresponds to the four-electron OER process. Γ is the assumed surface active-site density, which was taken as $1.0\times10^{15}$ sites $cm^{-2}{}_{ECSA}$ for a conservative atomic-scale surface-site approximation.

## 2 Supplementary figures and tables

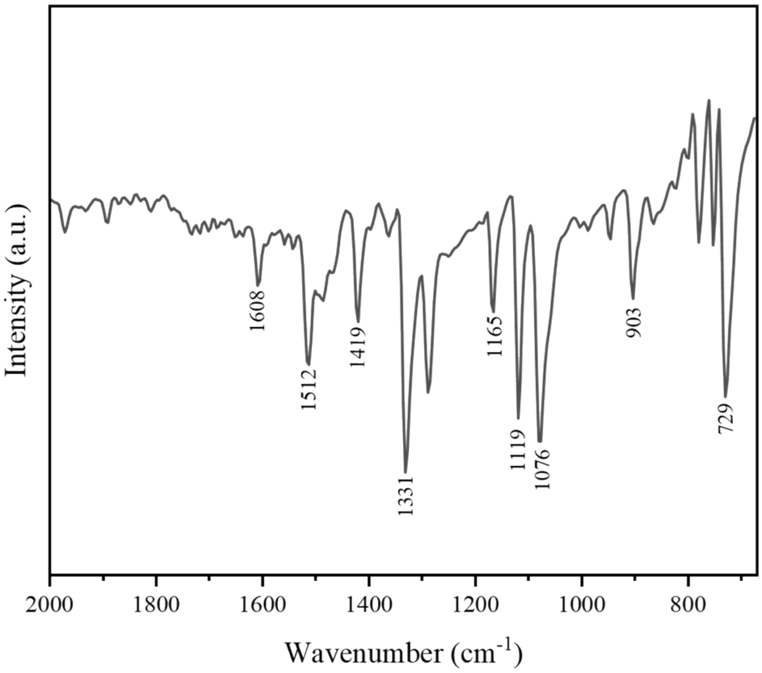


**Figure S1.** Micro-ATR-FTIR spectrum of the pristine FePc/Ni electrode prior to electrochemical activation.

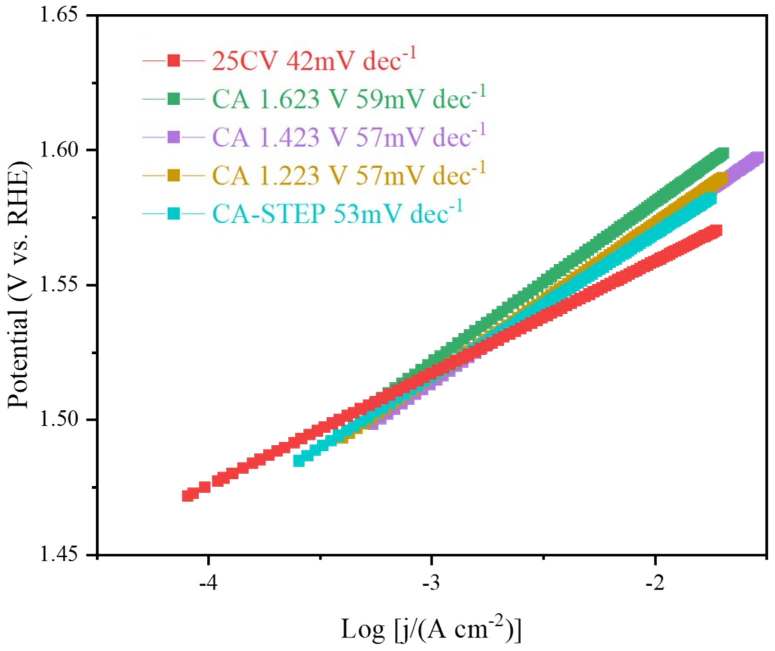


**Figure S2.** Tafel plots of FePc/Ni after different electrochemical activation protocols.

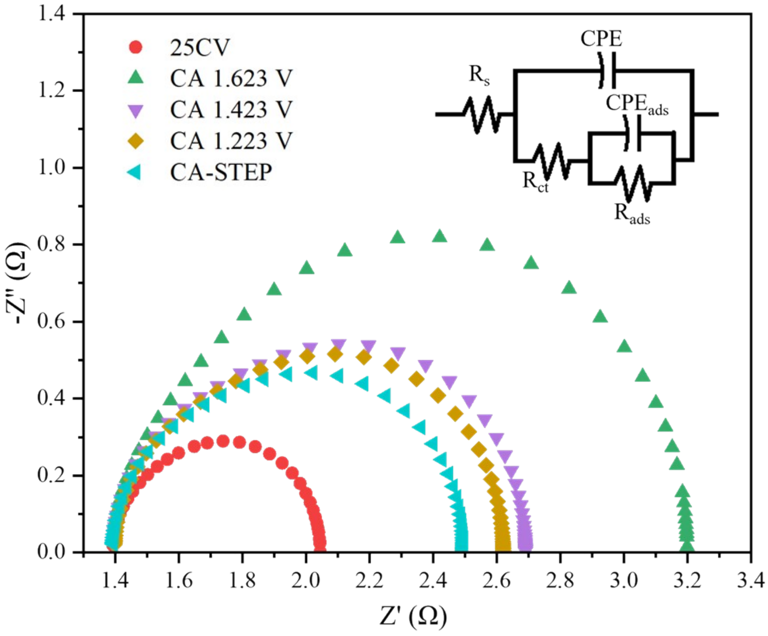


**Figure S3.** EIS Nyquist plots of FePc/Ni after different electrochemical activation protocols.

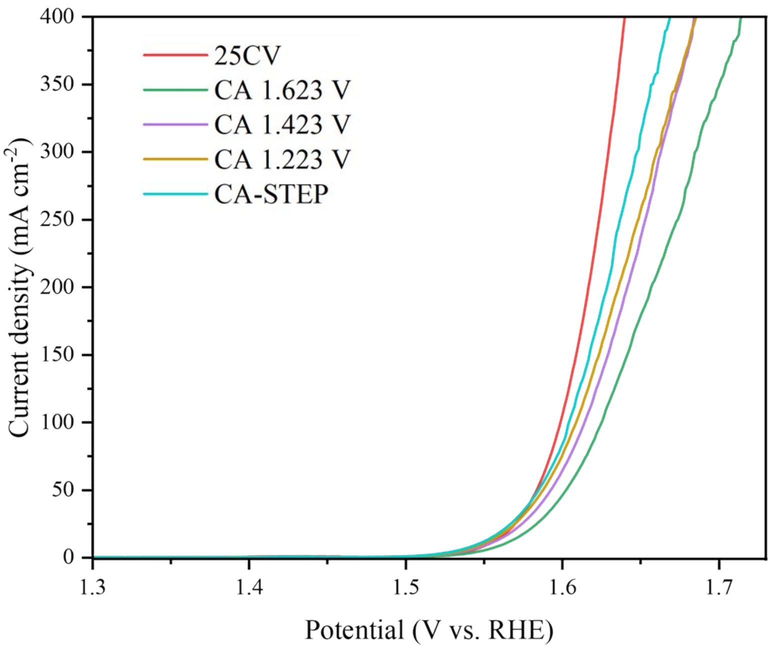


**Figure S4.** LSV curves of FePc/Ni after different electrochemical activation protocols.

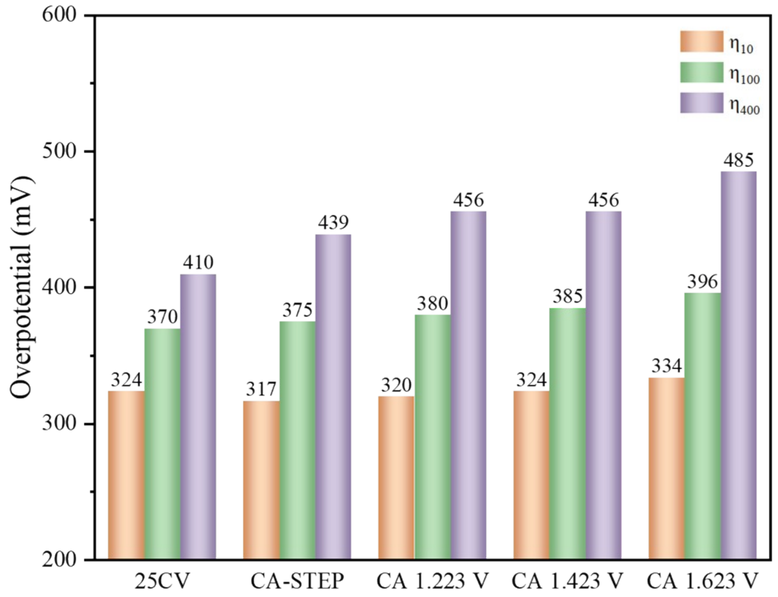


**Figure S5.** Overpotentials of FePc/Ni after different electrochemical activation protocols.

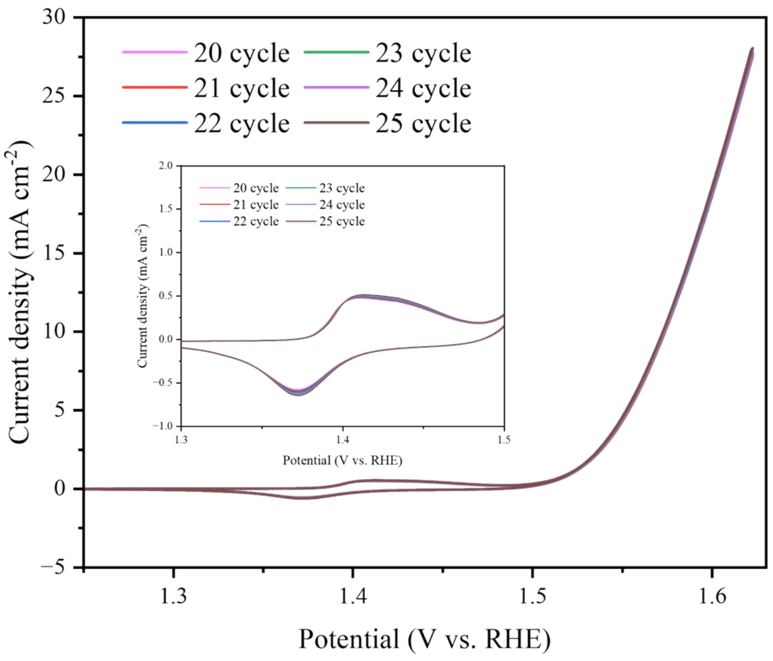


**Figure S6.** CV curves of FePc/Ni during activation (cycles 20-25).

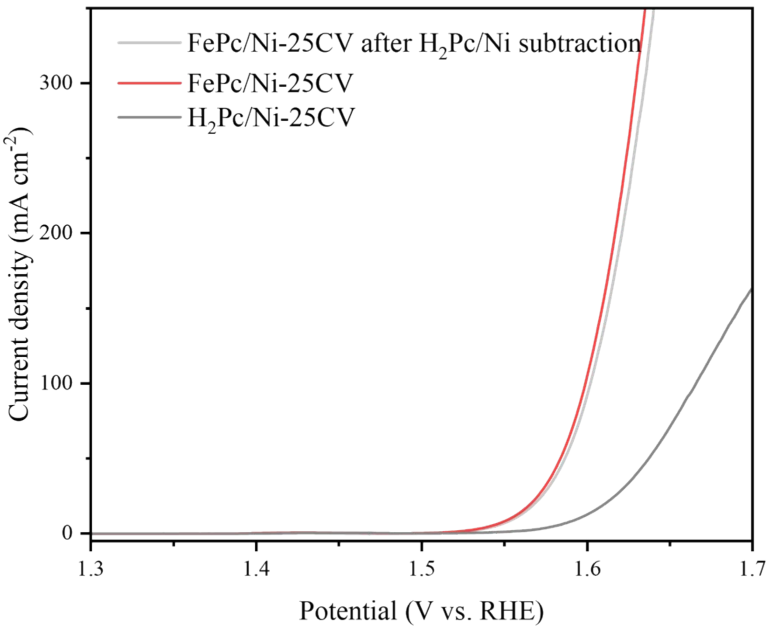


**Figure S7.** OER polarization curves of FePc/Ni-25CV, $H_2Pc$/Ni-25CV, and FePc/Ni-25CV after subtracting the $H_2Pc$/Ni-25CV current. The $H_2Pc$/Ni-25CV current density was subtracted from that of FePc/Ni-25CV at the same potential to estimate the possible contribution from Pc ligand-related oxidation.

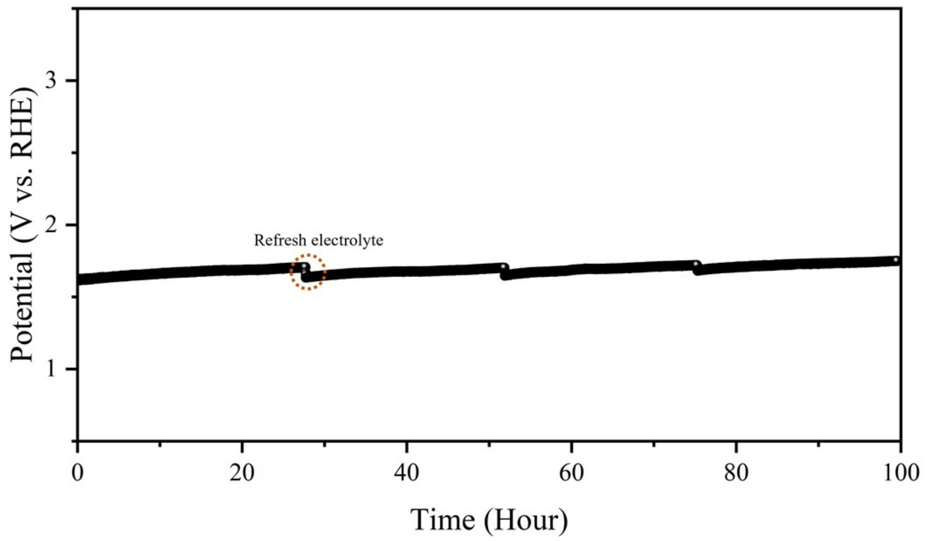


**Figure S8.** Stability of the FePc/Ni-25CV in 1 M KOH at 100 mA $cm^{-2}$.

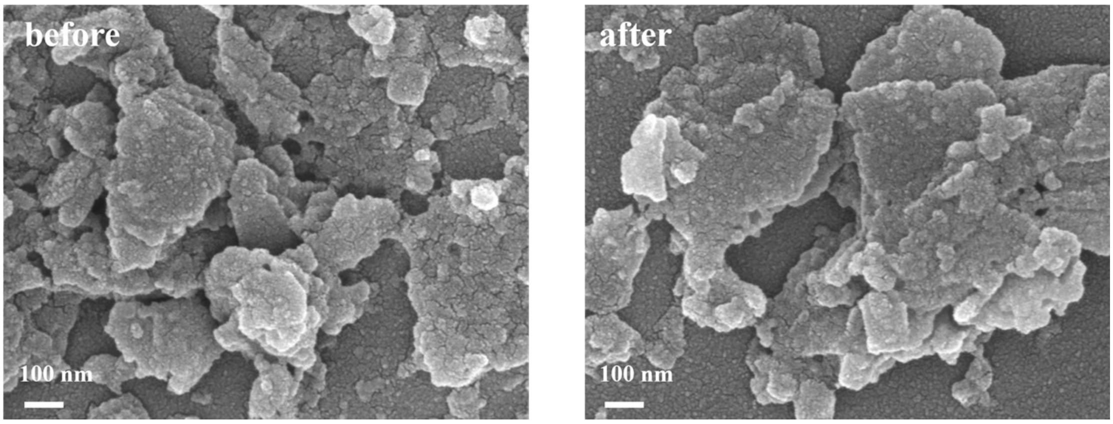


**Figure S9.** SEM images of FePc/Ni-25CV before and after the 100 mA $cm^{-2}$ chronopotentiometry test.

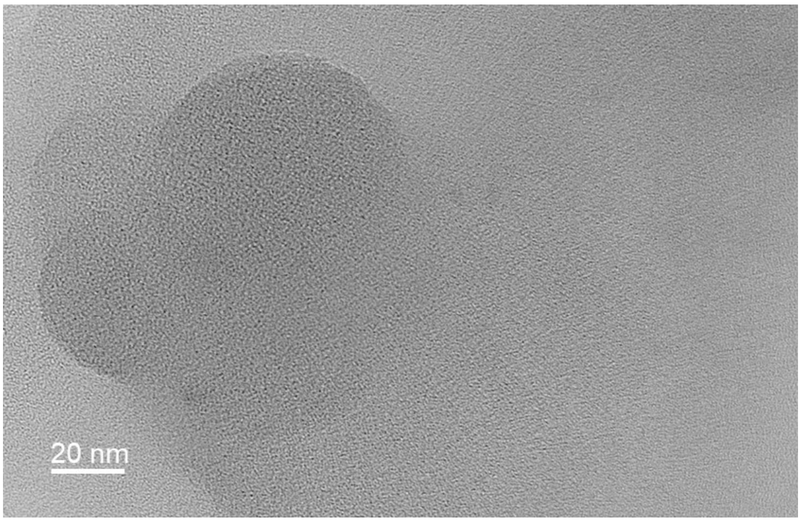


**Figure S10.** The TEM image of FePc/Ni after CV activation.

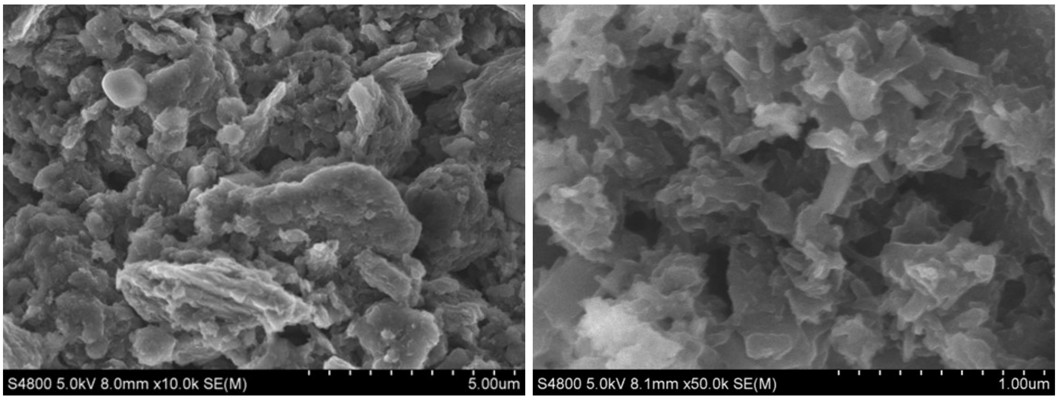


**Figure S11.** SEM images of the original FePc powder.

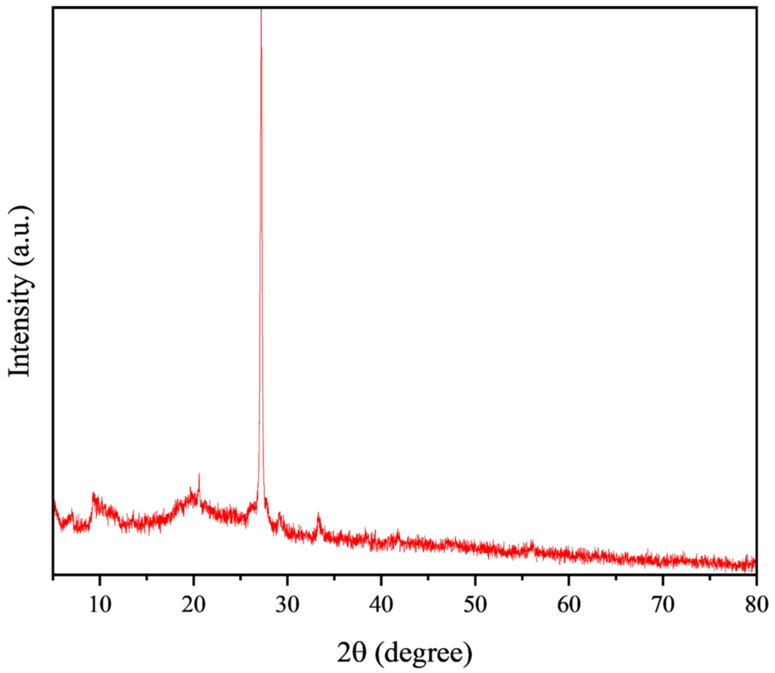


**Figure S12.** XRD pattern of the original FePc powder.

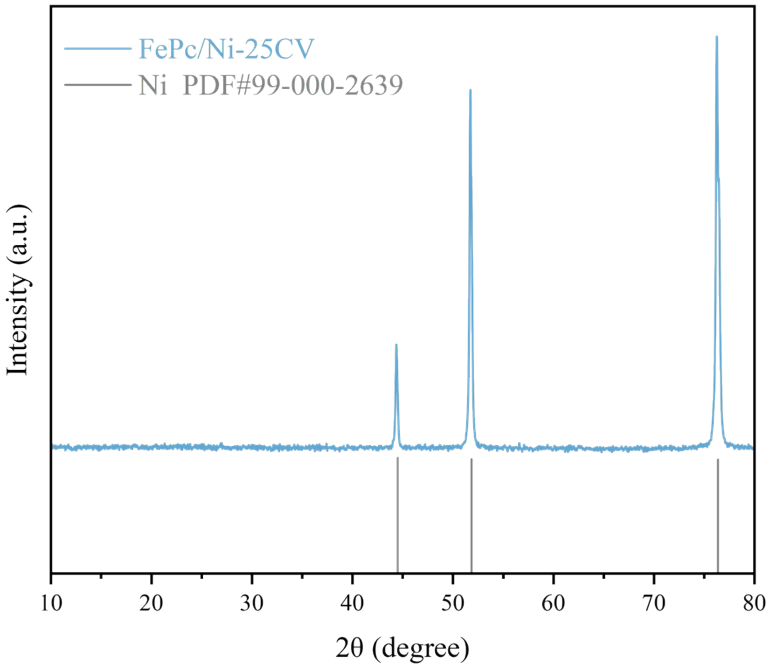


**Figure S13.** XRD pattern of the activated FePc/Ni-25CV electrode, together with the standard diffraction positions of metallic Ni (PDF#99-000-2639).

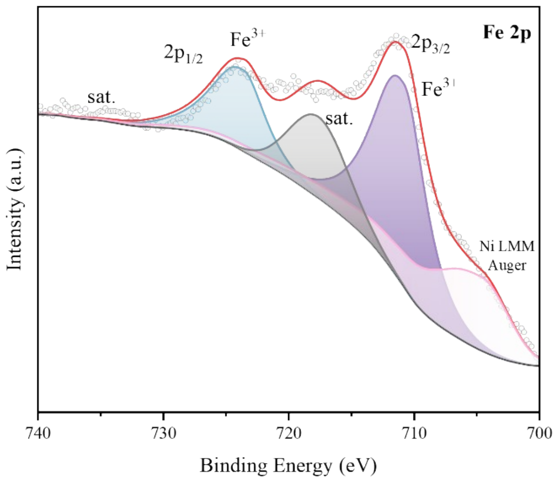


**Figure S14.** High-resolution Fe 2p XPS spectrum of FePc/Ni-25CV.

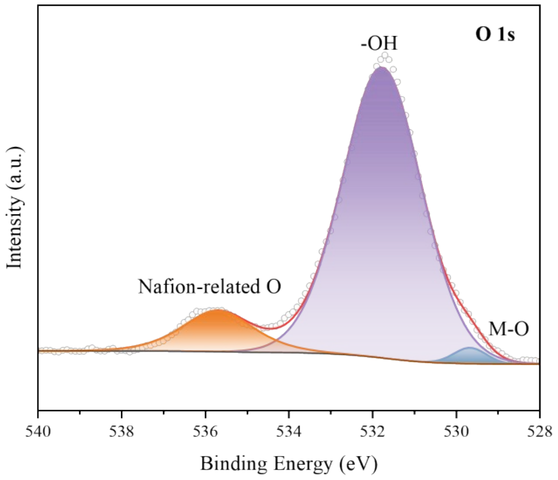


**Figure S15.** High-resolution O 1s XPS spectrum of FePc/Ni-25CV.

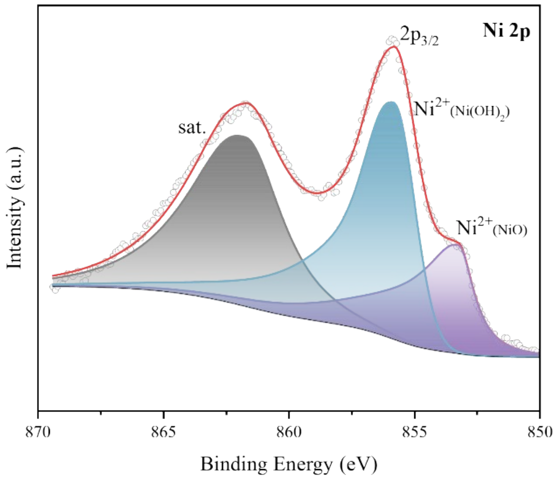


**Figure S16.** High-resolution Ni 2p XPS spectrum of FePc/Ni-25CV.

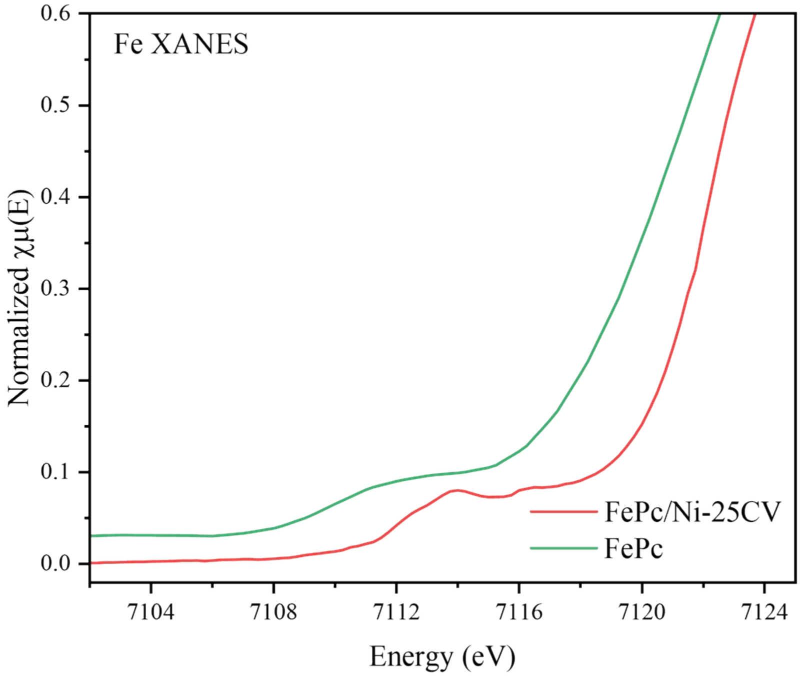


**Figure S17.** Enlarged view of the Fe K-edge XANES spectra in the region containing the pre-edge feature for FePc and FePc/Ni-25CV, showing the relative change of the pre-edge feature after activation.

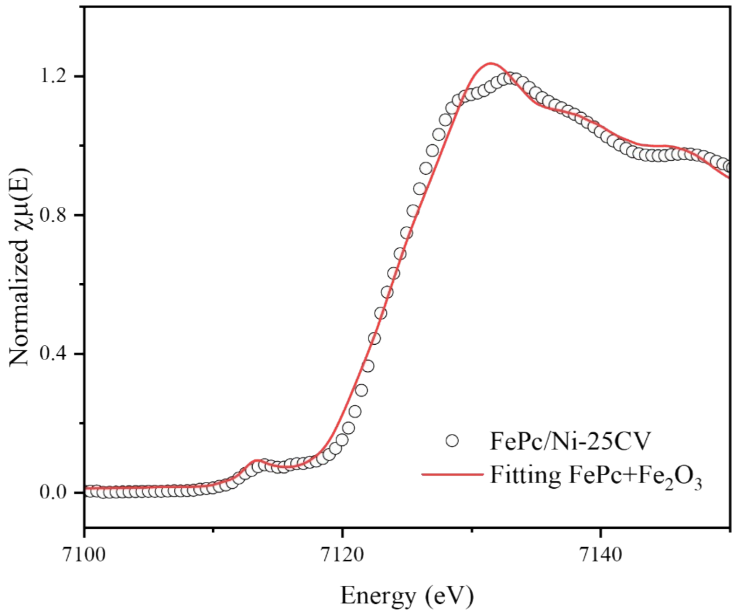


**Figure S18.** Fe K-edge XANES linear combination fitting of FePc/Ni-25CV using pristine FePc and $Fe_2O_3$ as reference compounds.

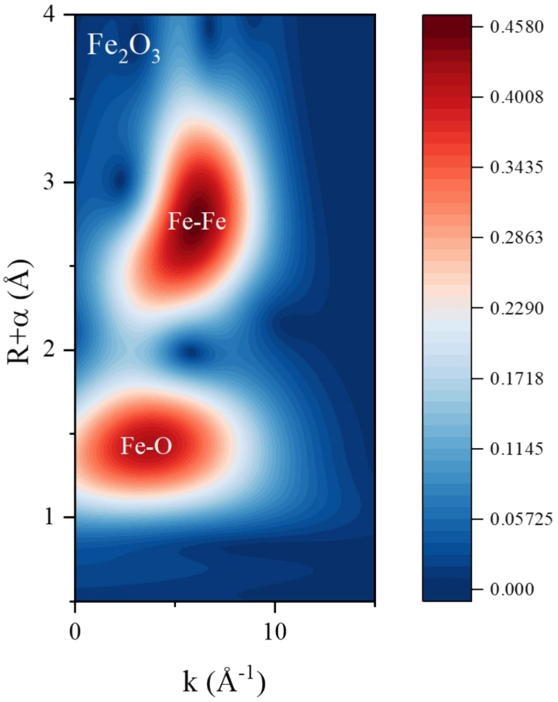


**Figure S19.** Wavelet transform plots of Fe K-edge EXAFS signals for $Fe_2O_3$.

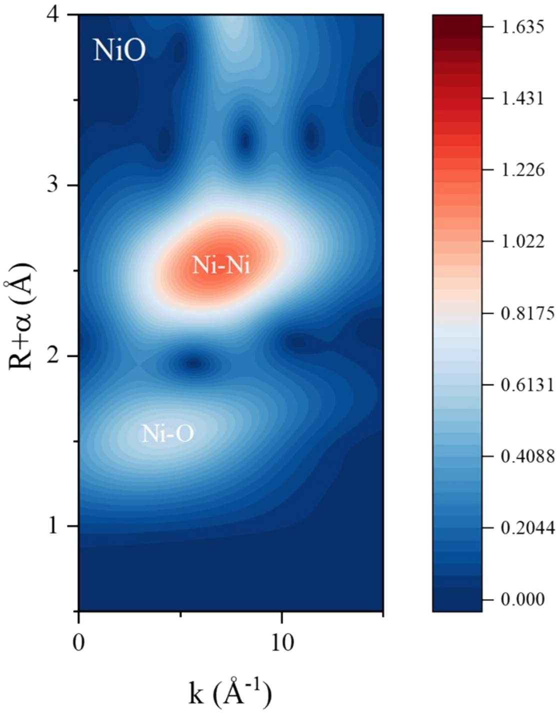


**Figure S20.** Wavelet transform plots of Ni K-edge EXAFS signals for NiO.

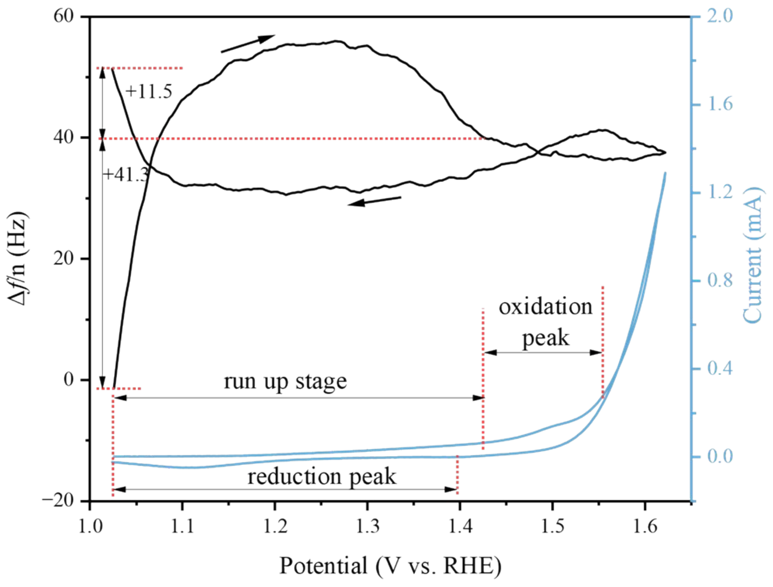


**Figure S21.** Current-potential (blue) and Δ*f*/n (black) during the 1st CV activation cycle of FePc/Ni.

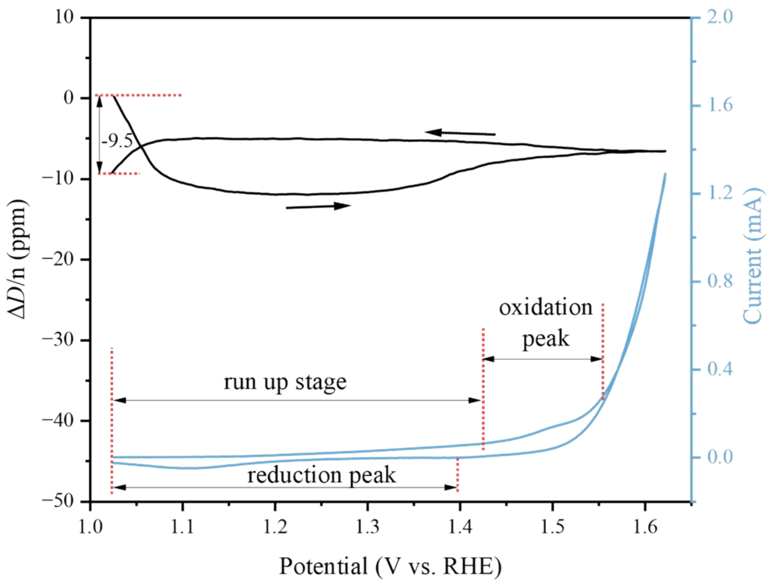


**Figure S22.** Current-potential (blue) and Δ*D*/n (black) during the 1st CV activation cycle of FePc/Ni.

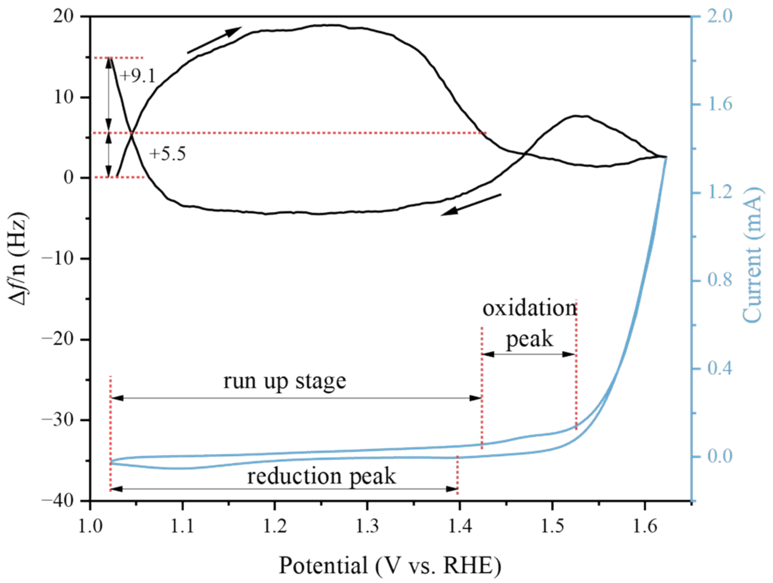


**Figure S23.** Current-potential (blue) and Δ*f*/n (black) during the 2nd CV activation cycle of FePc/Ni.

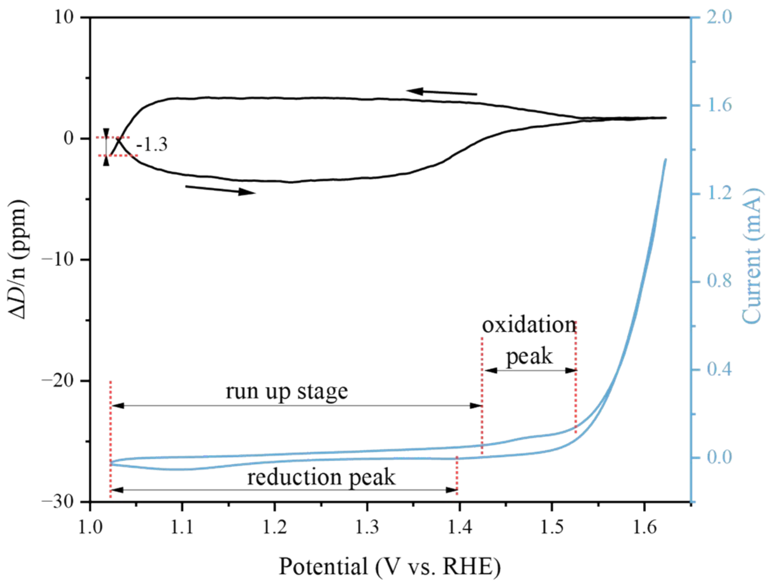


**Figure S24.** Current-potential (blue) and Δ*D*/n (black) during the 2nd CV activation cycle of FePc/Ni.

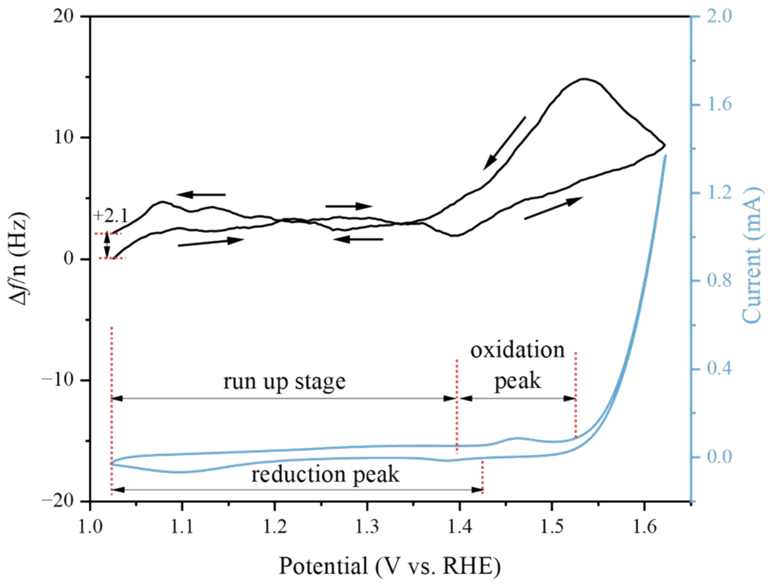


**Figure S25.** Current-potential (blue) and Δ*f*/n (black) during the 24th CV activation cycle of FePc/Ni.

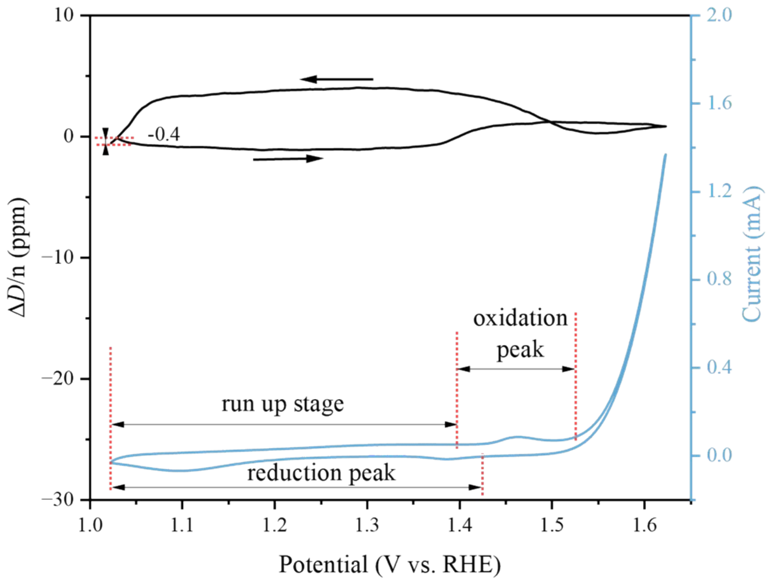


**Figure S26.** Current-potential (blue) and Δ*D*/n (black) during the 24th CV activation cycle of FePc/Ni.

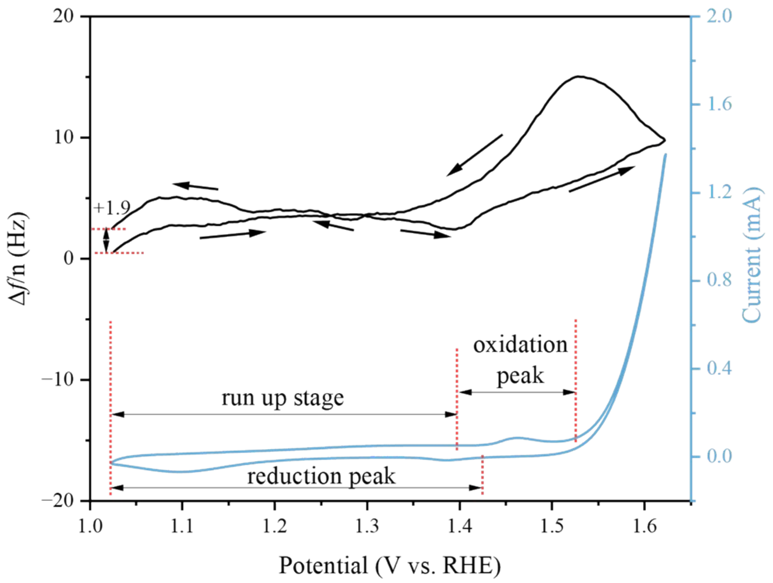


**Figure S27.** Current-potential (blue) and Δ*f*/n (black) during the 25th CV activation cycle of FePc/Ni.

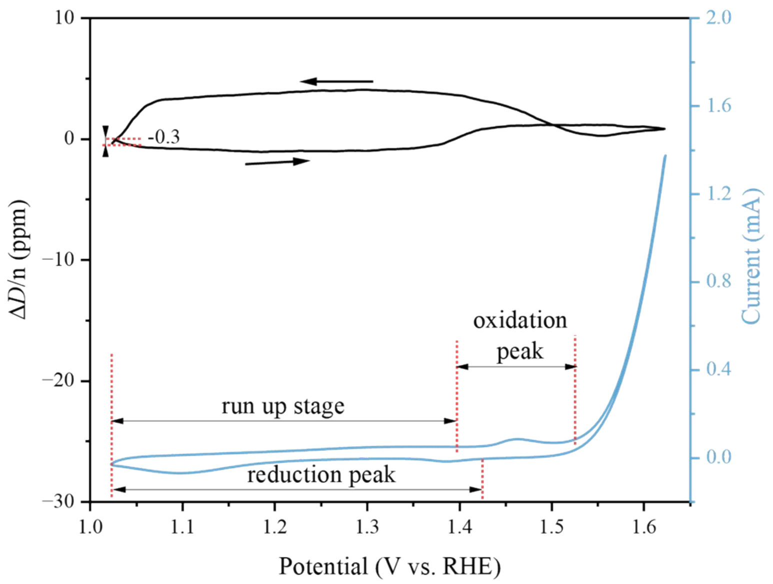


**Figure S28.** Current-potential (blue) and Δ*D*/n (black) during the 25th CV activation cycle of FePc/Ni.

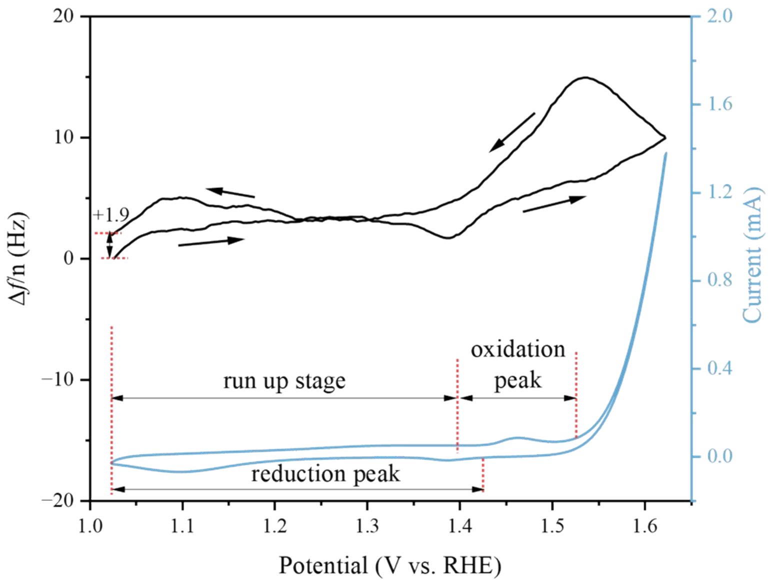


**Figure S29.** Current-potential (blue) and Δ*f*/n (black) during the 26th CV activation cycle of FePc/Ni.

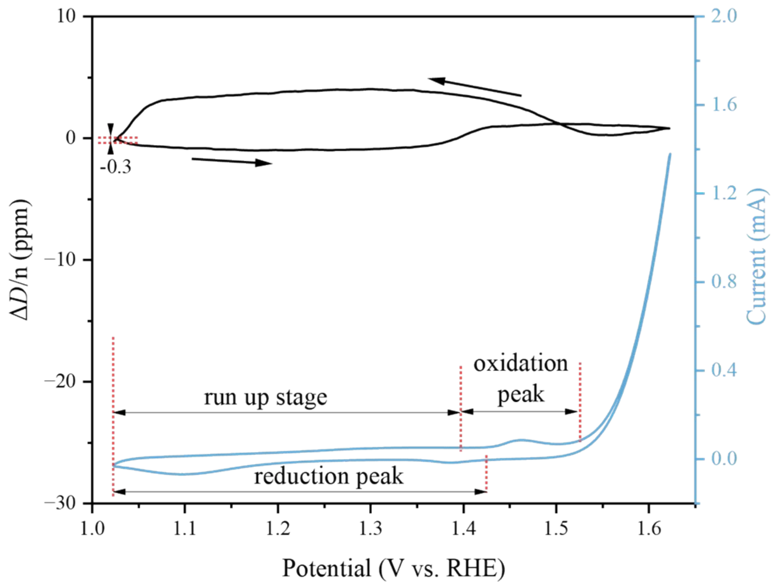


**Figure S30.** Current-potential (blue) and Δ*D*/n (black) during the 26th CV activation cycle of FePc/Ni.

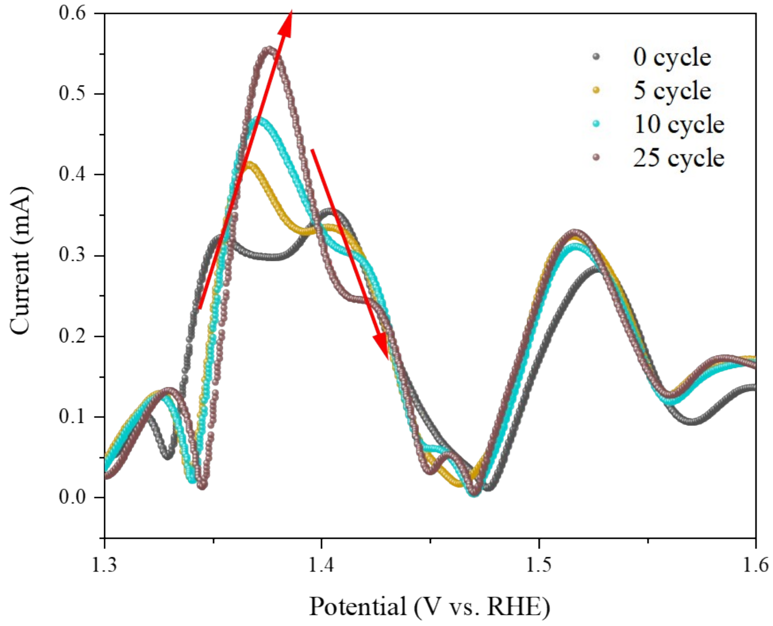


**Figure S31.** Third-harmonic FTacV responses of the FePc/Ni electrode recorded at different CV cycle numbers.

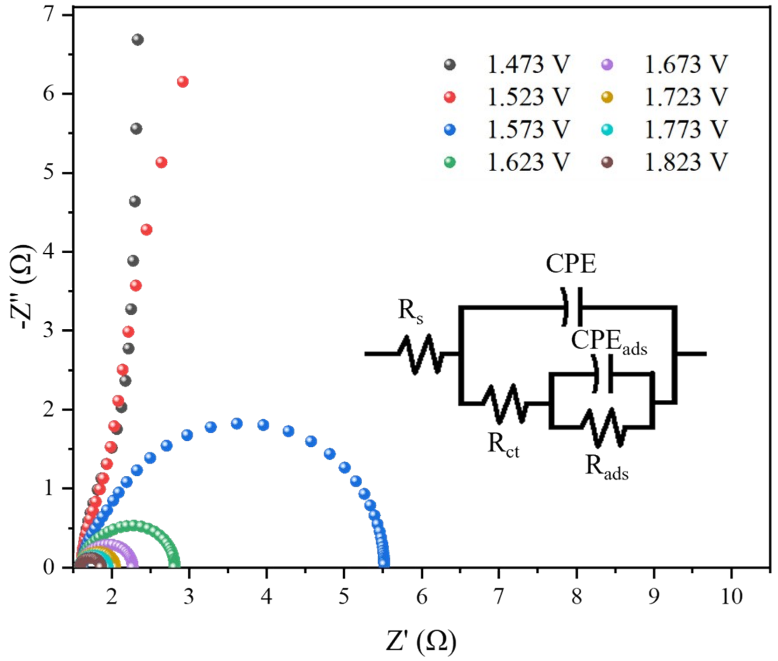


**Figure S32.** Nyquist plots for the original FePc/Ni at different applied potentials.

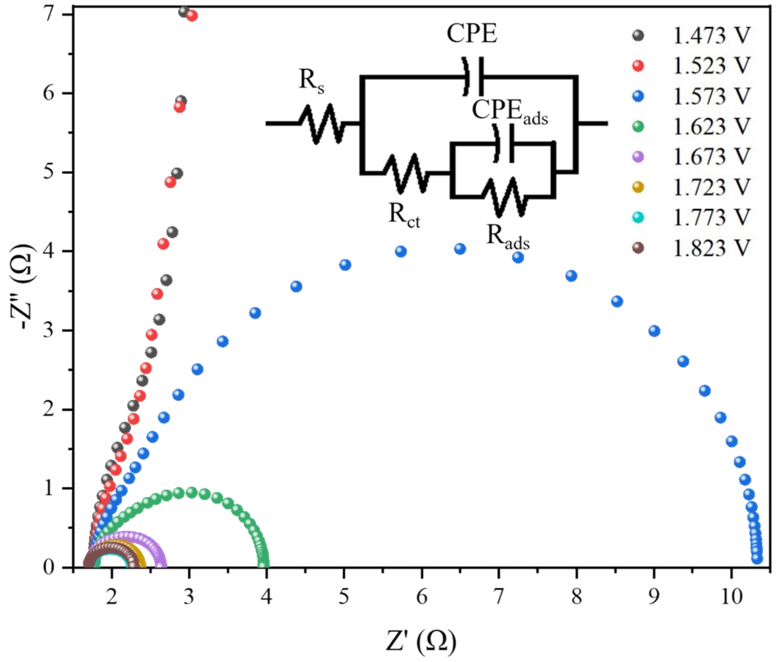


**Figure S33.** Nyquist plots for pure Ni at different applied potentials.

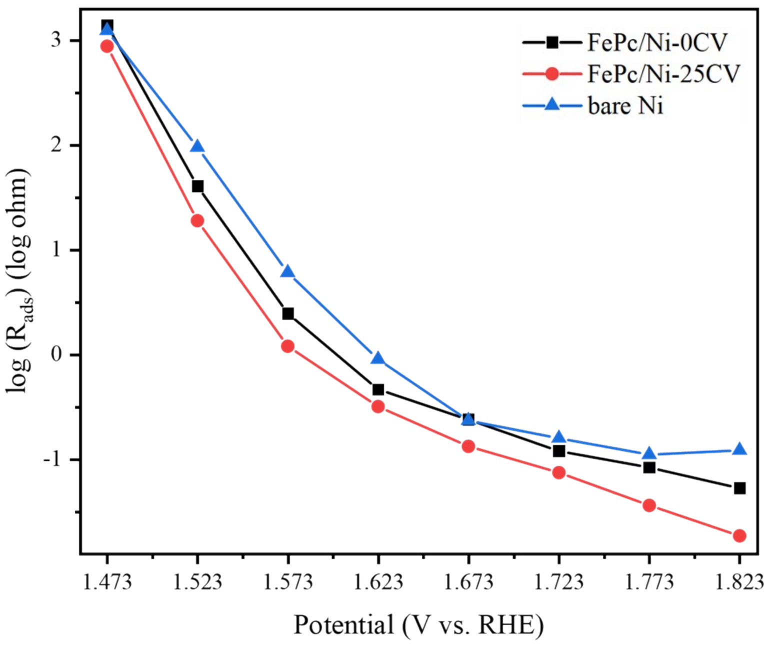


**Figure S34.** Potential-dependent $R_{ads}$ derived from potential-dependent EIS for FePc/Ni-0CV, FePc/Ni-25CV and bare Ni.

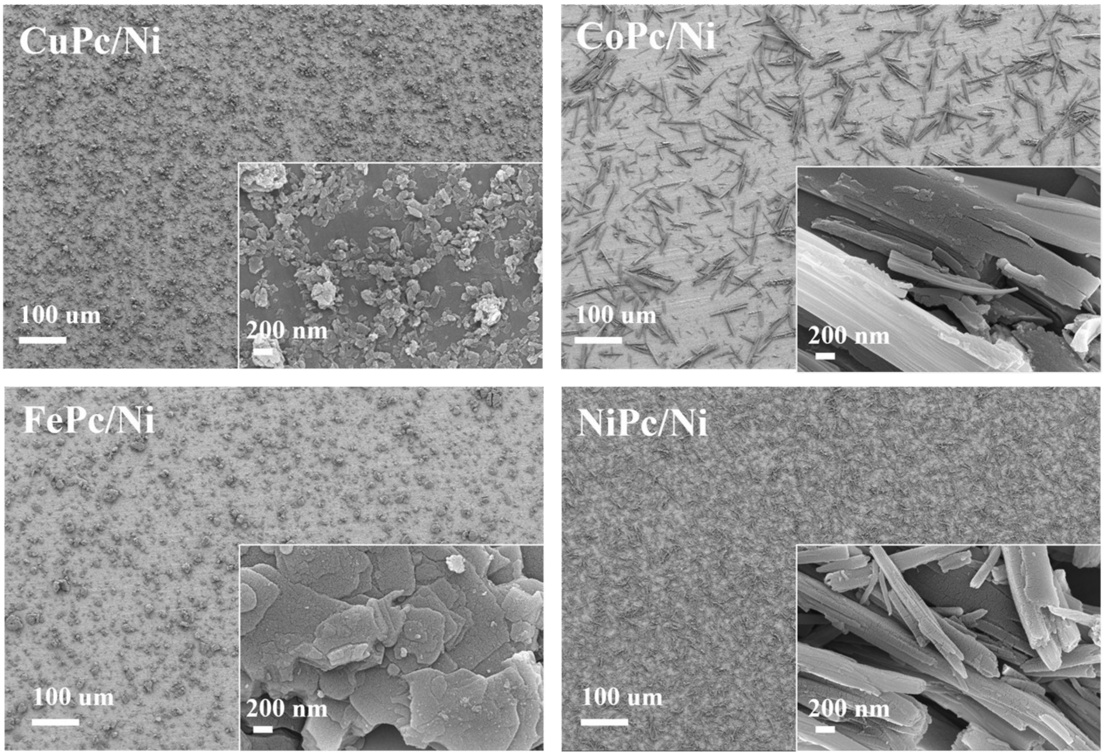


**Figure S35.** SEM images of the as-deposited $M_1$Pc/Ni electrodes.

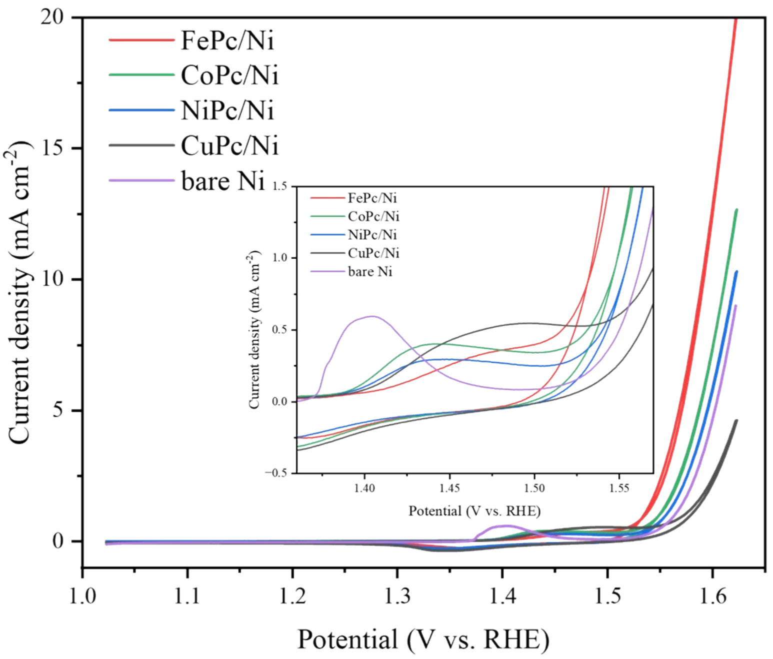


**Figure S36.** First-cycle CV responses of $M_1Pc/Ni$ and bare Ni electrodes. The inset shows an enlarged view of the oxidation-feature region to compare the initial electrochemical activation behaviour of the corresponding electrodes more clearly.

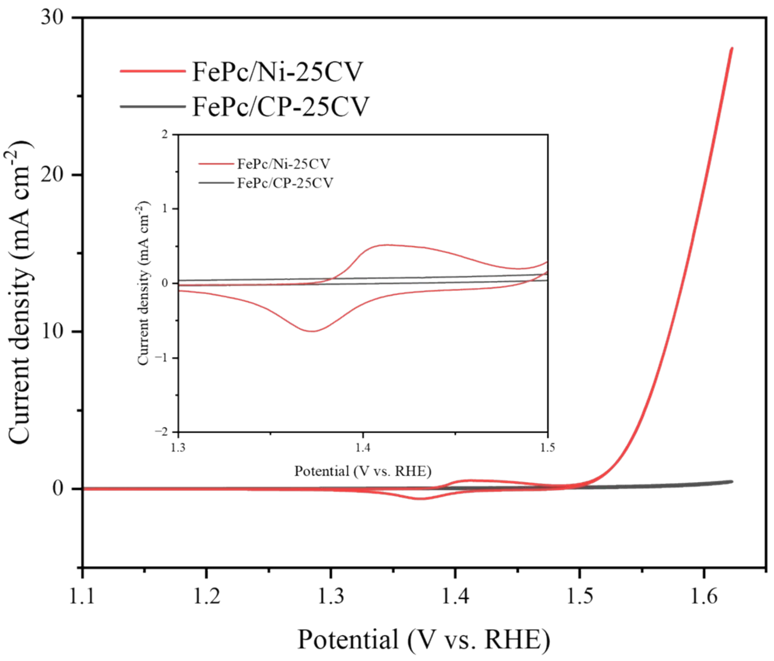


**Figure S37.** CV curves of FePc/Ni-25CV and FePc/CP-25CV. The inset shows the enlarged pre-OER redox region.

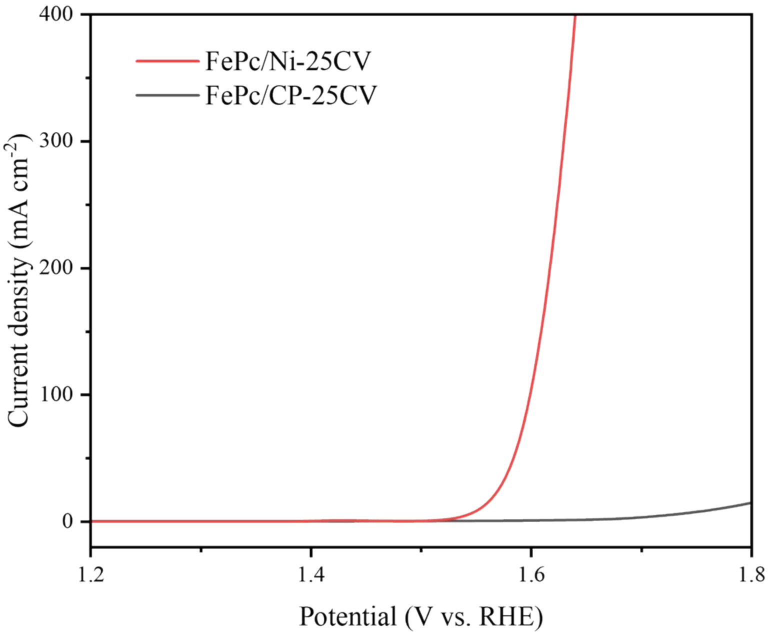


**Figure S38.** OER polarization curves of FePc/Ni-25CV and FePc/CP-25CV.

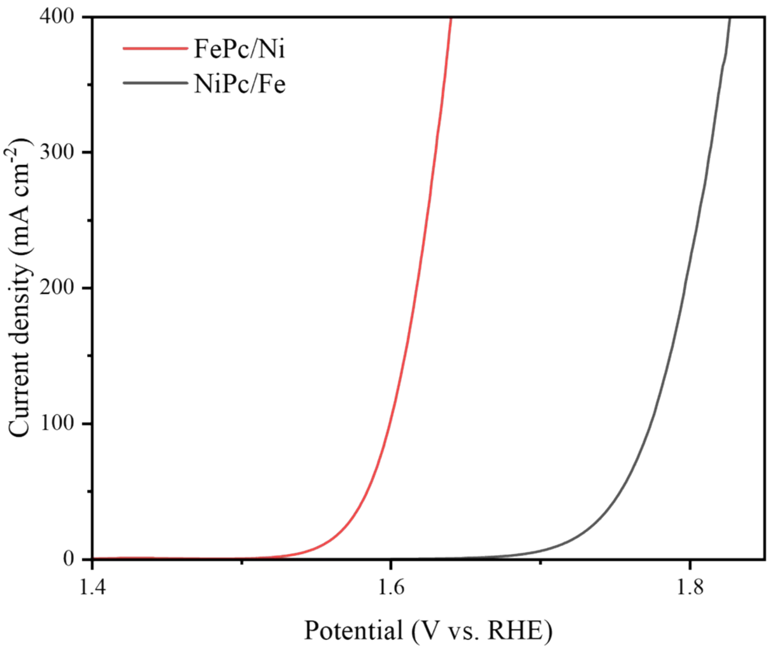


**Figure S39.** LSV curves of FePc/Ni and NiPc/Fe after CV activation.

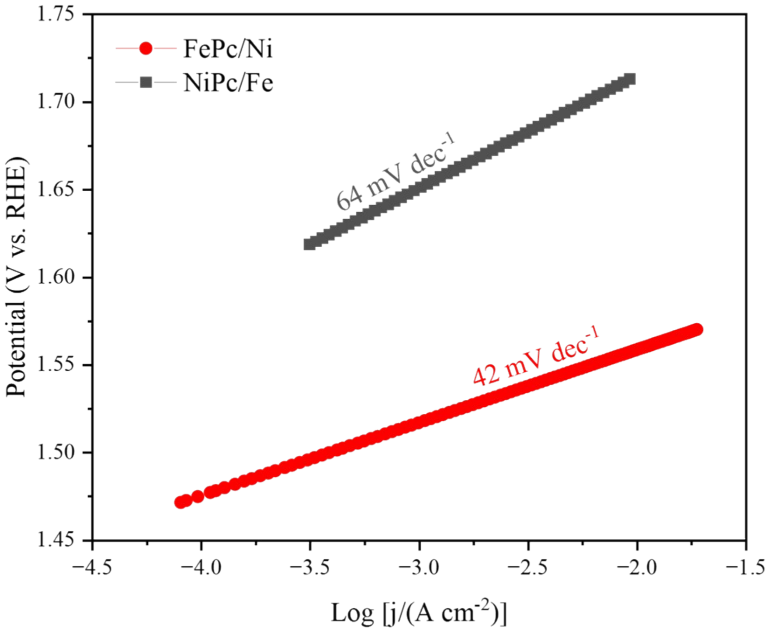


**Figure S40.** Tafel plots of FePc/Ni and NiPc/Fe after CV activation.

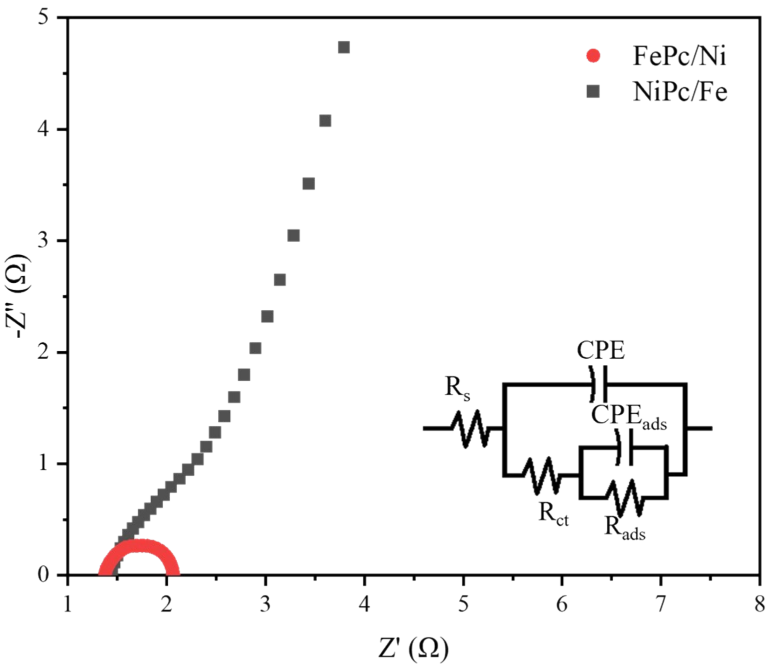


**Figure S41.** EIS Nyquist plots of FePc/Ni and NiPc/Fe after CV activation.

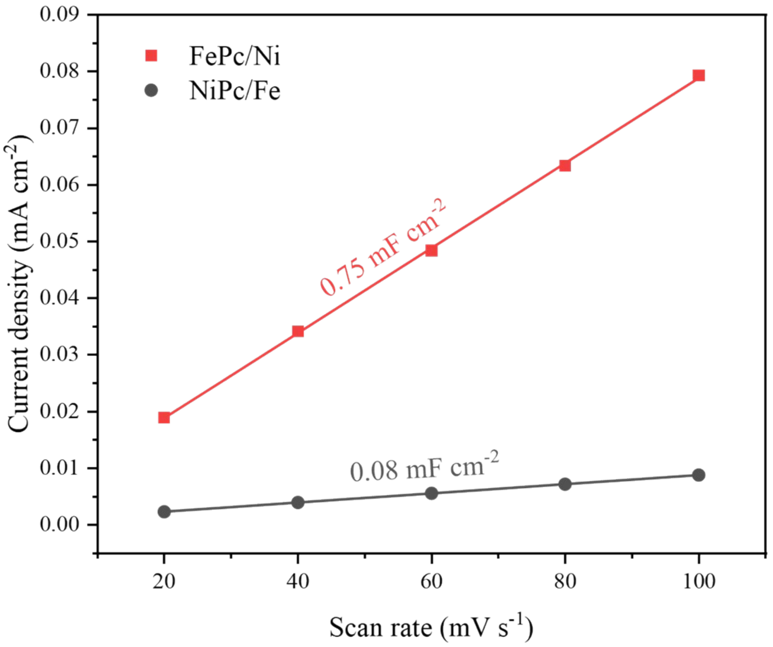


**Figure S42.** $C_{dl}$ fitting plots of FePc/Ni and NiPc/Fe after CV activation.

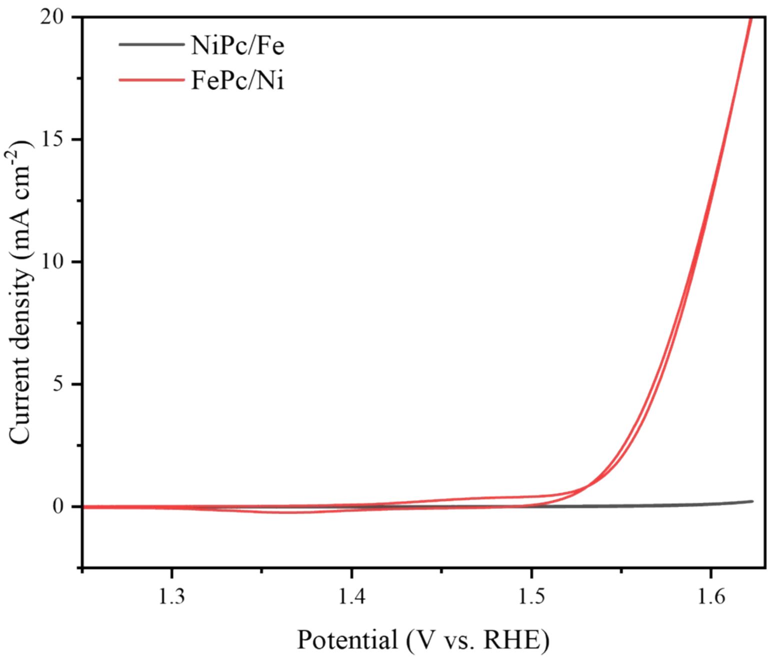


**Figure S43.** First-cycle CV curves of FePc/Ni and NiPc/Fe.

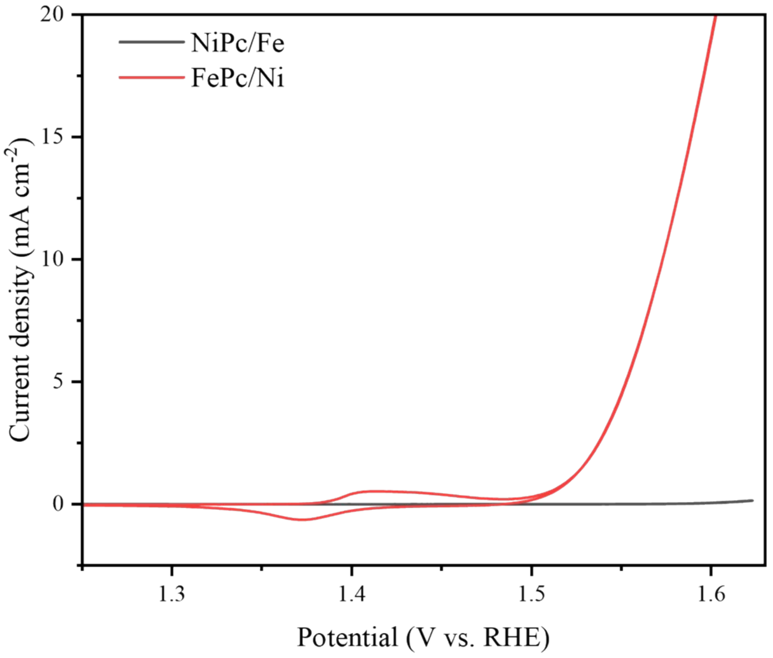


**Figure S44.** 25th-cycle CV curves of FePc/Ni and NiPc/Fe.

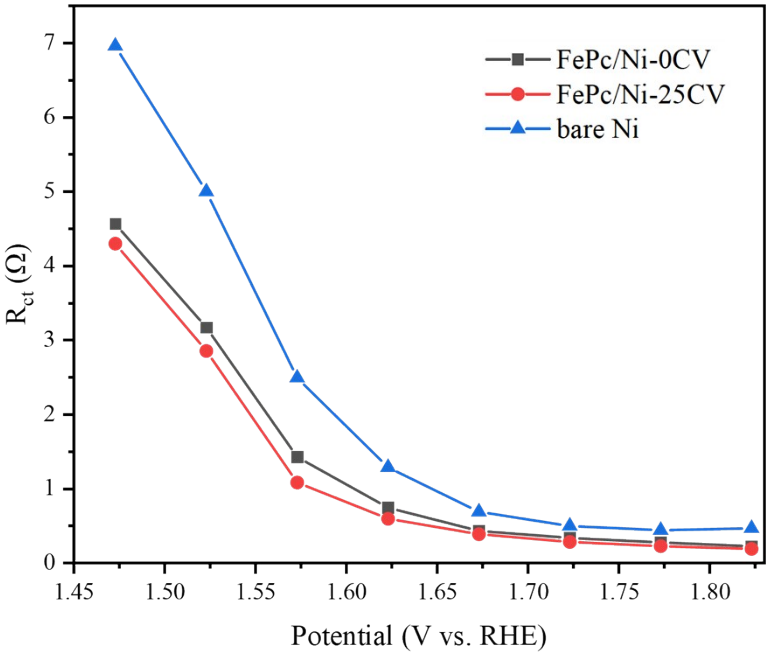


**Figure S45.** Potential-dependent $R_{ct}$ derived from potential-dependent EIS for FePc/Ni-0CV, FePc/Ni-25CV and bare Ni.

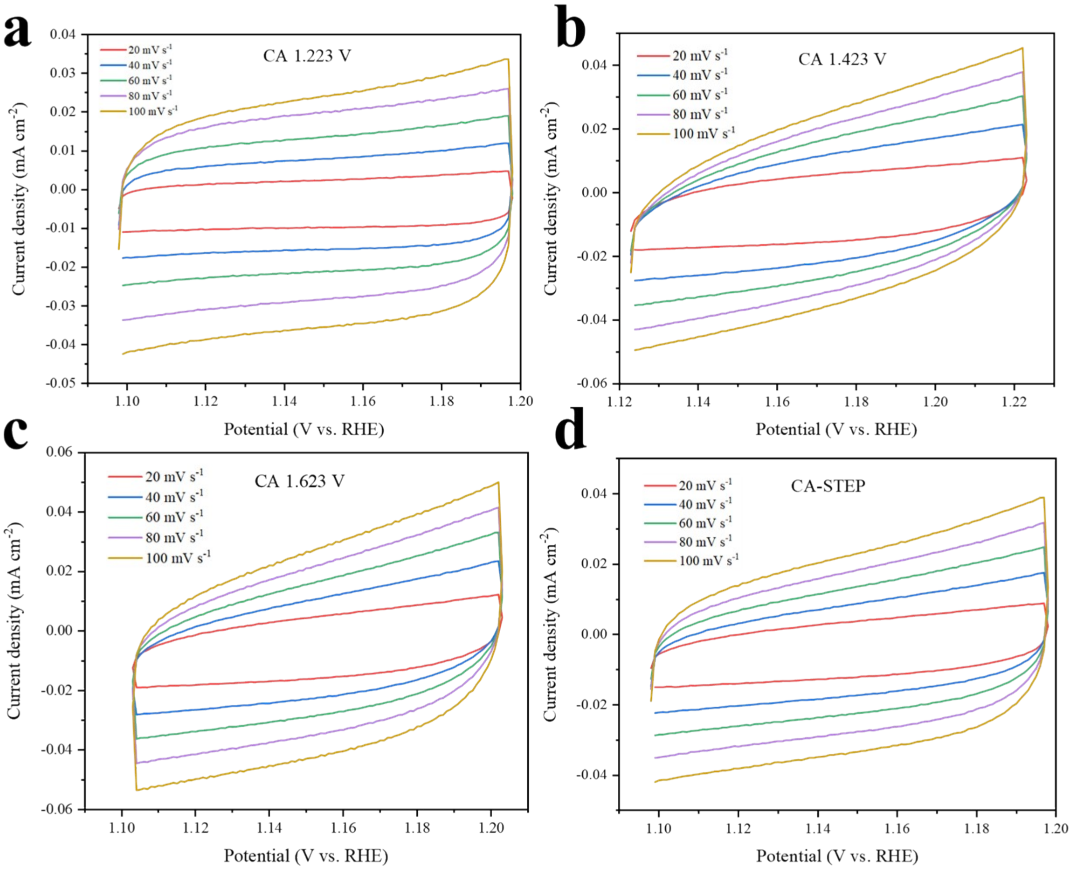


**Figure S46.** CV curves of FePc/Ni recorded at different scan rates for $C_{\mathrm{dl}}$ determination after different electrochemical activation protocols. (a) CA 1.223 V, (b) CA 1.423 V, (c) CA 1.623 V and (d) CA-STEP.

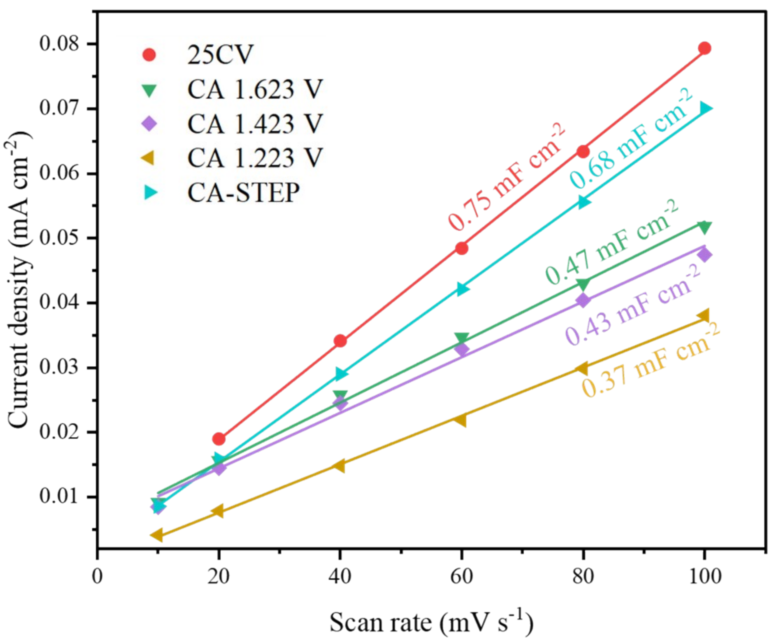


**Figure S47.** $C_{dl}$ fitting plots of FePc/Ni after different electrochemical activation protocols.

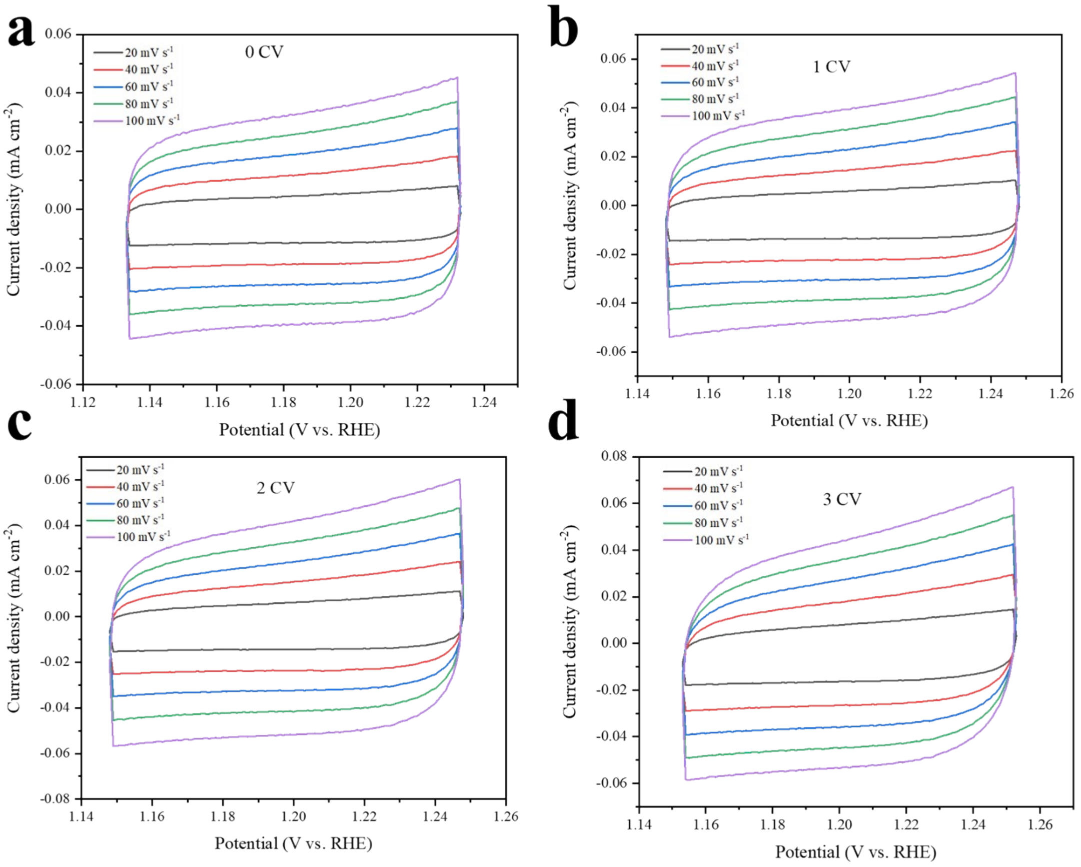


**Figure S48.** Scan-rate-dependent CV curves of FePc/Ni after selected CV cycle numbers for $C_{\mathrm{dl}}$ determination. (a) Pristine (before activation), (b) 1st, (c) 2nd and (d) 3rd cycles.

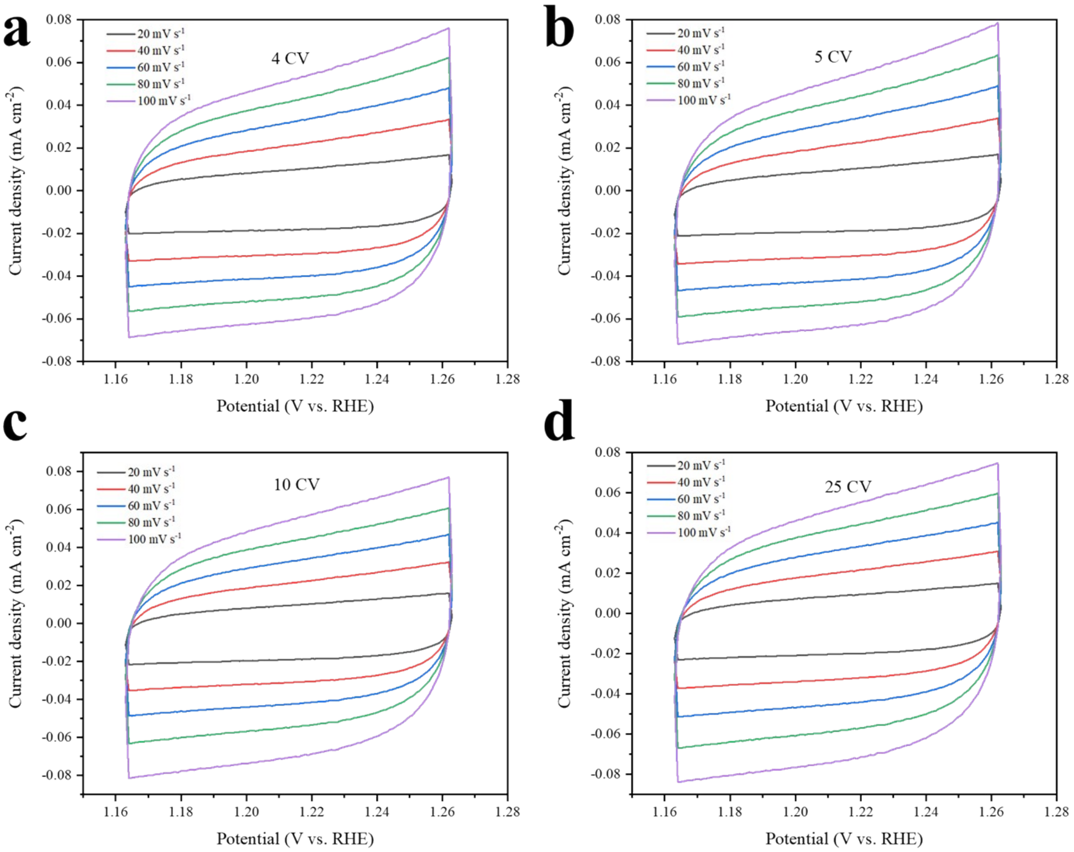


**Figure S49.** Scan-rate-dependent CV curves of FePc/Ni after selected CV cycle numbers for $C_{dl}$ determination. (a) 4th, (b) 5th, (c) 10th and (d) 25th cycles.

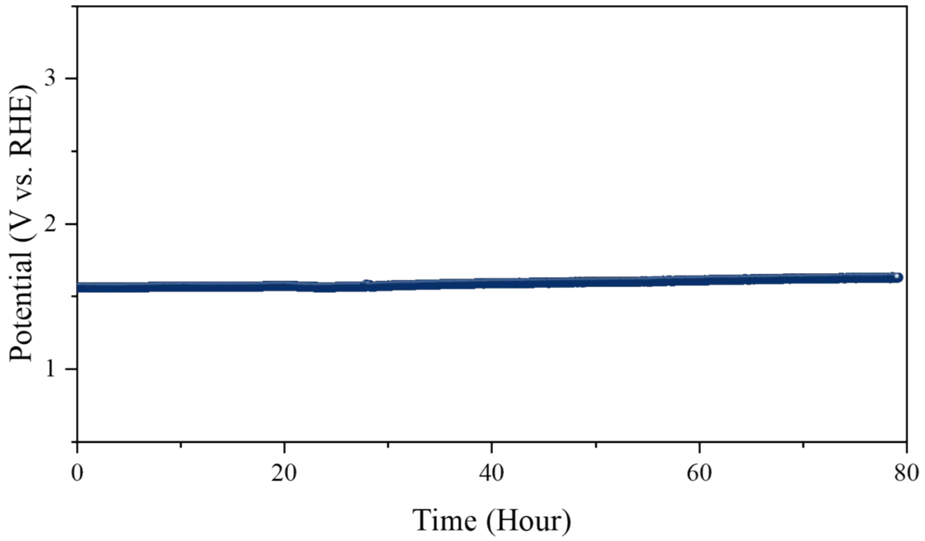


**Figure S50.** Stability of the FePc/Ni-25CV in 1 M KOH at 10 mA $cm^{-2}$.

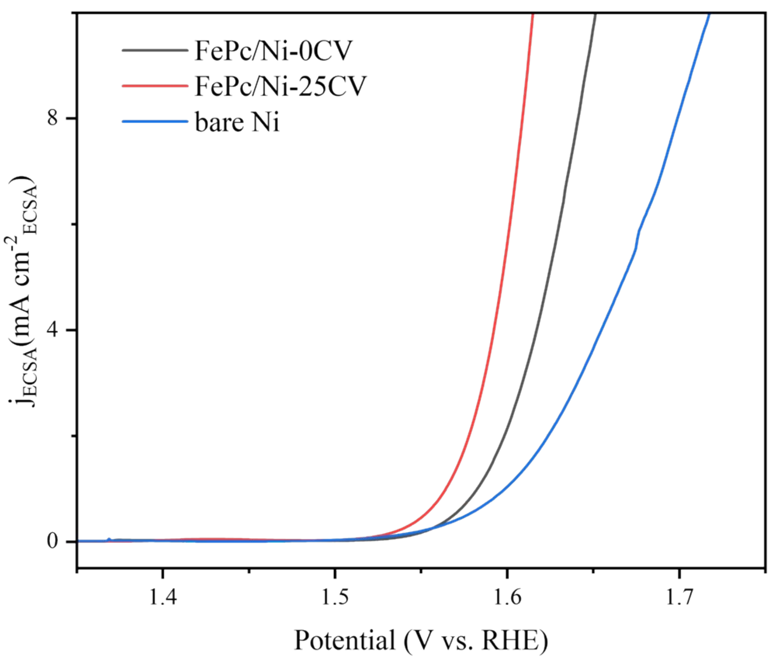


**Figure S51.** ECSA-normalized OER polarization curves of FePc/Ni-0CV, FePc/Ni-25CV, and bare Ni.

**Table S1.** Summary of OER performance metrics of FePc/Ni after different electrochemical activation protocols.

| **Activation protocols** | **Overpotential** | | | **Tafel slope** | $R_{ct}$ | $C_{dl}$ |
|---|---|---|---|---|---|---|
| | $\eta_{10}$ | $\eta_{100}$ | $\eta_{400}$ | | | |
| 25CV | 324 | 370 | 410 | 42 | 0.4421 | 0.75 |
| CA-STEP | 317 | 375 | 439 | 53 | 0.7032 | 0.67 |
| CA 1.223 V | 320 | 380 | 456 | 57 | 0.7450 | 0.38 |
| CA 1.423 V | 324 | 385 | 456 | 57 | 0.7883 | 0.41 |
| CA 1.623 V | 334 | 396 | 485 | 59 | 0.8947 | 0.45 |

**Table S2.** Electrochemical OER performance metrics of FePc/Ni during CV activation.

| **Cycle** | **Overpotential** | | **Tafel slope** | $R_{ct}$ | $R_{ads}$ | $C_{dl}$ |
|---|---|---|---|---|---|---|
| | $\eta_{10}$ | $\eta_{100}$ | | | | |
| 0 | 352 | 419 | 65 | 0.6132 | 0.5349 | 0.43 |
| 1 | 337 | 393 | 49 | 0.5657 | 0.4152 | 0.52 |
| 2 | 332 | 385 | 48 | 0.5128 | 0.3166 | 0.56 |
| 3 | 329 | 380 | 47 | 0.4912 | 0.2592 | 0.58 |
| 4 | 328 | 376 | 47 | 0.4889 | 0.2489 | 0.67 |
| 5 | 328 | 377 | 46 | 0.4807 | 0.2374 | 0.70 |
| 10 | 327 | 374 | 44 | 0.4453 | 0.2169 | 0.74 |
| 25 | 324 | 370 | 42 | 0.4421 | 0.2109 | 0.75 |

**Table S3.** Comparison of representative Fe/Ni-related OER catalysts reported in the literature.

| Catalyst | Support | $\eta_{10}$ (mV) | Tafel slope (mV $dec^{-1}$) | TOF ($s^{-1}$) | Reference |
|---|---|---|---|---|---|
| FePc/Ni-25CV | Ni foil | 324@$\eta_{10}$ | 42 | 0.331 | This work |
| $Ni_3S_2$/Ni | Ni foil | 341@$\eta_{10}$ | 87 | - | [7] |
| FeS/Fe | Fe foil | 477@$\eta_{10}$ | 143 | - | [7] |
| $Fe_{2.5}$@NiNPs | Glassy carbon | 341@$\eta_{10}$ | 70 | - | [8] |
| $Fe(PO_3)_2/Ni_2P$ | Ni foam | 177@$\eta_{10}$ | 51.9 | 0.12@$\eta$=300 mV | [9] |
| MIL-53(Fe–Ni)/NF-2200Gs | Ni foam | 174@$\eta_{10}$ | 58 | 0.25@$\eta$=211 mV | [10] |
| $Ir_1$/Ni LDH-T | Ni foam | 228@$\eta_{10}$ | 41 | 0.12@$\eta$=300 mV | [11] |
| $Ir_1$/Ni LDH-V | Ni foam | 301@$\eta_{10}$ | 62 | 0.006@$\eta$=300 mV | [11] |
| Ni LDH | Ni foam | 321@$\eta_{10}$ | 59 | 0.003@$\eta$=300 mV | [11] |
| $NiFe_{0.5}$Sn-A | carbon cloth | 260@$\eta_{10}$ | 50 | 0.194@$\eta$=300 mV | [12] |
| NiFe LDH/FeMoO | Ni foam | 316@$\eta_{1000}$ | 48.9 | 0.14@$\eta$=300 mV | [13] |
| NiFe LDH | Ni foam | 399@$\eta_{1000}$ | 70.7 | 0.056@$\eta$=300 mV | [13] |
| FePc@NF | Ni foam | 289@$\eta_{10}$ | 49.9 | 0.04@$\eta$=300 mV | [14] |
| NiPc@NF | Ni foam | 352@$\eta_{10}$ | 67.2 | 0.002@$\eta$=300 mV | [14] |
| $\beta$-$Ni(OH)_2$ nanoplates | Ni foam | 340@$\eta_{10}$ | 69 | 0.182@$\eta$=300 mV | [15] |

**Table S4.** XPS-derived near-surface elemental composition of FePc/Ni-25CV.

| Fe (at%) | Ni (at%) | C (at%) | N (at%) | O (at%) |
|---|---|---|---|---|
| 3.78 | 4.09 | 61.56 | 14.80 | 15.77 |

**Table S5.** Fe K-edge edge positions of different samples.

| Sample | Edge Position ($E_{edge}$) (*e*V) |
|---|---|
| Fe foil | 7117.88 |
| FePc | 7121.22 |
| $Fe_3O_4$ | 7121.35 |
| $Fe_2O_3$ | 7122.19 |
| FePc/Ni-25CV | 7122.80 |

**Table S6.** Linear combination fitting results of the Fe K-edge XANES spectrum of FePc/Ni-25CV.

| Sample | Standard | Weight | R factor |
|---|---|---|---|
| FePc/Ni-25CV | $Fe_2O_3$ | 0.805±0.035 | 0.004 |
| | FePc | 0.195±0.045 | |

**Table S7.** Ni K-edge edge positions of different samples.

| **Sample** | **Edge Position ($E_{edge}$) (*e*V)** |
|---|---|
| Ni foil | 8331.90 |
| NiO | 8337.97 |
| FePc/Ni-25CV | 8332.64 |

**Table S8.** Comparison of the key OER performance metrics of $M_1Pc/M_2$ electrodes after standardized 25-cycle CV activation.

| Electrode | $\eta_{10}$ (mV) | $\eta_{100}$ (mV) | Tafel slope (mV dec$^{-1}$) | $R_{ct}$ (Ω) |
|---|---|---|---|---|
| FePc/Ni | 324 | 370 | 42 | 0.44 |
| CoPc/Ni | 349 | 448 | 80 | 2.15 |
| NiPc/Ni | 373 | 479 | 89 | 2.79 |
| CuPc/Ni | 425 | 603 | 95 | 4.75 |
| FePc/Co | 394 | 442 | 83 | 3.52 |
| FePc/Fe | 499 | 565 | 135 | 8.56 |
| FePc/Cu | 583 | 725 | 147 | 184 |

**References**


1 B. Ravel and M. Newville, *J. Synchrot. Radiat.*, 2005, **12**, 537-541.

2 H. Xu, S.-J. Xie, C. Lv, J.-T. Li, Y. Zhou and S.-G. Sun, *J. Mater. Chem. A*, 2023, **11**, 10277-10286.

3 F. Dong, H. Duan, Z. Lin, H. Yuan, M. Ju, X. Du, J. Gao, J. Yu and S. Yang, *Appl. Catal. B Environ.*, 2024, **340**, 123242.

4 S. Sun, T. Wang, R. Liu, Z. Sun, X. Hao, Y. Wang, P. Cheng, L. Shi, C. Zhang and X. Zhou, *Ultrason. Sonochem.*, 2024, **109**, 107027.

5 H. Dau, P. Liebisch and M. Haumann, *Analytical and Bioanalytical Chemistry*, 2003, **376**, 562-583.

6 A. Roy, S. Kumar, A. Guilherme Buzanich, C. Prinz, E. Götz, A. Retzmann, T. Hickel, B. Bhattacharya and F. Emmerling, *Adv. Mater.*, 2024, **36**, 2408114.

7 C.-Z. Yuan, Z.-T. Sun, Y.-F. Jiang, Z.-K. Yang, N. Jiang, Z.-W. Zhao, U. Y. Qazi, W.-H. Zhang and A.-W. Xu, *Small*, 2017, **13**, 1604161.

8 D. D. Huguet, V. Varela-Izquierdo, J. Cervera Martin, B. Chaudret, K. Soulantica, D. R. Sánchez, M. D. d. l. B. Sanchez and C. Godard, *Int. J. Hydrogen Energy*, 2025, **184**, 151868.

9 H. Zhou, F. Yu, J. Sun, R. He, S. Chen, C.-W. Chu and Z. Ren, *Proceedings of the National Academy of Sciences*, 2017, **114**, 5607-5611.

10 Y. Miao, Q. Huang, D. Wen, D. Xie, B. Huang, D. Lin, C. Xu, W. Zeng and F. Xie, *RSC Advances*, 2023, **13**, 4249-4254.

11 J. Wei, H. Tang, L. Sheng, R. Wang, M. Fan, J. Wan, Y. Wu, Z. Zhang, S. Zhou and J. Zeng, *Nat. Commun.*, 2024, **15**, 559.

12 M. Chen, S. Lu, X.-Z. Fu and J.-L. Luo, *Advanced Science*, 2020, **7**, 1903777.

13 S. Liu, M. Sun, L. Dai, S. Wang, Z. Yu, N. Li, F. Chen, Y. Wei, Y. Shen, C. Zhao and M. Wang, *Nat. Commun.*, 2026, **17**, 2135.

14 X. Meng, P. Yu and M. Zhang, *Molecules*, 2024, **29**, 4272.

15 N. Kim, D. Lim, Y. Choi, S. E. Shim and S.-H. Baeck, *Electrochim. Acta*, 2019, **324**, 134868.
